# Predicting opioid dependence from electronic health records with machine learning

**DOI:** 10.1186/s13040-019-0193-0

**Published:** 2019-01-29

**Authors:** Randall J. Ellis, Zichen Wang, Nicholas Genes, Avi Ma’ayan

**Affiliations:** 1 Department of Pharmacological Sciences, Mount Sinai Center for Bioinformatics, Icahn School of Medicine at Mount Sinai, New York, NY 10029 USA; 2 Department of Emergency Medicine, Mount Sinai Hospital, New York, NY 10029 USA

**Keywords:** Opioid epidemic, Opioid dependence, Electronic health records, Electronic medical records, Machine learning, Artificial intelligence

## Abstract

**Background:**

The opioid epidemic in the United States is averaging over 100 deaths per day due to overdose. The effectiveness of opioids as pain treatments, and the drug-seeking behavior of opioid addicts, leads physicians in the United States to issue over 200 million opioid prescriptions every year. To better understand the biomedical profile of opioid-dependent patients, we analyzed information from electronic health records (EHR) including lab tests, vital signs, medical procedures, prescriptions, and other data from millions of patients to predict opioid substance dependence.

**Results:**

We trained a machine learning model to classify patients by likelihood of having a diagnosis of substance dependence using EHR data from patients diagnosed with substance dependence, along with control patients with no history of substance-related conditions, matched by age, gender, and status of HIV, hepatitis C, and sickle cell disease. The top machine learning classifier using all features achieved a mean area under the receiver operating characteristic (AUROC) curve of ~ 92%, and analysis of the model uncovered associations between basic clinical factors and substance dependence. Additionally, diagnoses, prescriptions, and procedures prior to the diagnoses of substance dependence were analyzed to elucidate the clinical profile of substance-dependent patients, relative to controls.

**Conclusions:**

The predictive model may hold utility for identifying patients at risk of developing dependence, risk of overdose, and opioid-seeking patients that report other symptoms in their visits to the emergency room.

**Electronic supplementary material:**

The online version of this article (10.1186/s13040-019-0193-0) contains supplementary material, which is available to authorized users.

## Introduction

In a highly visible report it was described how drug overdose deaths have substantially increased in the United States from 2010 to 2015 [1]. The estimated societal costs of prescription opioid overdoses, abuse, and dependence in the United States in 2013 totaled $78.5 billion [2]. The challenges for physicians combating the opioid epidemic include: 1) Determining which patients are at risk of developing opioid dependence when prescribed these medications for conventional pain treatment; 2) Determining which patients known to be addicted to opioids are most at risk of opioid overdose; and 3) Identifying drug-seeking patients who visit the Emergency Department (ED) for the secondary gain of obtaining an opioid prescription. Strategies to identify drug-seeking patients rely mostly on checking Prescription Drug Monitoring Programs (PDMPs) [3], examining past clinical perceptions (clinical gestalt), or exam findings such as withdrawal symptoms [4, 5]. Urine toxicology tests can detect opioid metabolites, but these tests are prone to false positives and negatives, and opioid metabolites only remain present in the urine for a short period [6].

Previous studies of biomedical variables predictive of opioid misuse and abuse have unraveled several salient factors, including chronic opioid prescriptions, history of psychiatric illness, non-opioid substance disorders, having a family member diagnosed with an opioid use disorder, the use of multiple pharmacies to fill prescriptions, having hepatitis C, and tobacco addiction [7–10]. These studies are based on various types of data, including pharmacy prescriptions, insurance claims, vital signs, and medical notes from electronic health records (EHR). For example, Ciesielski et al. [7] and Rice et al. [8], in two separate studies, used pharmacy and insurance claims information from over half a million patients to construct a multivariate logistic regression model to predict likelihood of opioid abuse. Similarly, Cochran et al. [9] and Dufour et al. [10] analyzed insurance claims databases to identify variables with predictive power to classify opioid use disorder patients. In a related study, Hylan et al. [11] tracked for four years 2752 patients that received chronic opioid treatment for their pain condition. To determine and predict opioid misuse, Hylan et al. also utilized natural language processing to analyze clinicians’ notes. All these past studies point to few common clinical factors that contribute to opioid pathology. Their observations support that the construction of predictive models of opioid misuse and abuse based on prior knowledge about the patient is feasible. So far, no prior work examined the predictive value of biological measures from standard lab tests for opioid misuse and abuse. In addition, all prior studies used either univariate statistics or multivariate linear models to discern associations between opioid misuse diagnosis and other clinical variables.

Finding a clinically objective signature of opioid abuse would assist physicians in offering the proper treatment to those patients who attempt to hide their addiction for other clinical conditions. Such a signature will be a composite biomarker that can be detected by machine learning methods. EHR systems have proliferated in the past decade, and are increasingly used to perform predictive diagnosis with non-linear machine learning methods [12–14]. EHR data include demographics, diagnoses, laboratory tests, vital signs, clinical notes, prescriptions, and procedures data. Examples of previous predictive studies that utilized EHR systems implementing machine learning methods include predicting the incidence of cardiovascular disease in patients with severe schizophrenia, bipolar disorder, or other non-organic psychosis [15]; length of hospital stay and time to readmission based on Research Domain Criteria in psychiatric patients [16]; unplanned readmission after discharge [17]; in-hospital mortality [18]; patient physiological age [19]; and many more. Here we describe the application of a machine learning classifier to predict substance dependence based on lab tests and vital signs using patient data derived from the Mount Sinai Medical Center (MSMC) EHR system. The lab tests and vital signs that are found to be the most useful in distinguishing substance dependent patients from controls were identified. Furthermore, the substance dependent population was clinically phenotyped by the over-representation of their diagnoses, prescriptions, and procedures during the five years prior to their first diagnosis of substance dependence.

## Methods

### Constructing the case and control populations

The MSMC EHR (Epic Systems, Verona, WI) data were organized into a de-identified collection. 42,825,357 diagnoses from the EHR were queried to find all patients with diagnoses belonging to the 304. * family of the International Classification of Diseases (ICD-9) codes (12,112 cases), referring to various forms of substance dependence [20]. Patients were excluded if their first 304.* diagnosis was made before they turned 20 years of age to avoid patients who were born with substance dependence or acquired substance dependence during childhood or adolescence. This filtering reduced the number of cases to 11,573. Lab tests and vital signs were obtained for all patients within a 20-day window around their 304 diagnosis. Initially, this analysis produced 873 types of lab tests and 51 types of vital signs. To construct a control population, the requirements were that all lab tests and vital signs are from patients older than 20 years, have no history of diagnoses in ICD-9 code families 291–293: alcohol- and drug-induced mental disorders and withdrawal; 303–305: alcohol/drug dependence, abuse; and 964.9–978.0: poisoning by psychoactive substances. This filtering step left 828,062 patients as controls.

Modified z-scores [21] were calculated for all lab tests and vital signs for the cases and controls. Some values in the EHR data are mistakenly entered, for example, a height of 2376 ft was observed. To remove outliers from the case and control populations, all lab tests and vital signs with modified z-scores below −2.5 or above 2.5 were removed. Additionally, percentages below 0 or above 100 were removed. After removing outliers, we retained 9518 cases and 707,015 controls.

**Fig. 1:**
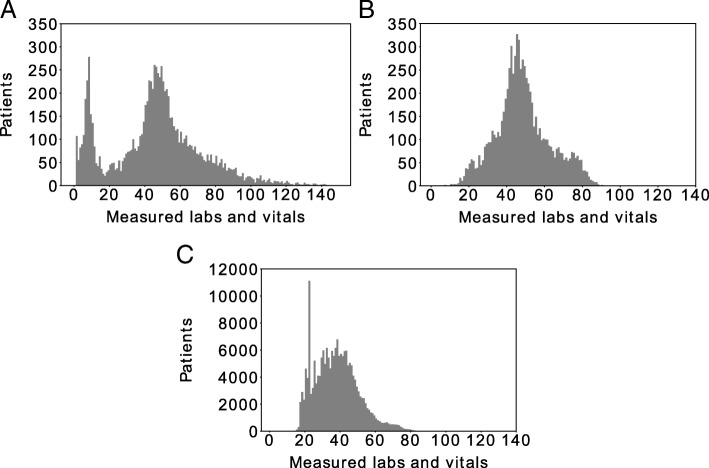
**a** Distribution of tests (labs and vitals) per case before filtering. **b** Distribution of tests (labs and vitals) per case after removing patients with less than 17 tests and tests with 90% or greater missing values. **c** Distribution of tests (labs and vitals) per control after removing tests with 90% or greater missing values

The distribution of lab tests and vital signs per case contains a large portion of cases with fewer than 20 lab tests and vital signs, while the rest of the lab tests and vital signs per case distribution forms an approximate bell curve (Fig. 1a). To retain only cases without sparse data points, cases with fewer than 17 lab tests and vital signs were excluded. This yielded 7797 cases with 889 unique lab tests (838) and vital signs (51); and 191,476 controls. Similarly, lab tests and vital signs with 90% or more missing values across all cases were removed. After these two steps, the distribution of lab tests and vital signs per case was approximately normal (Fig. 1b), leaving a case population of 7797 patients and 109 lab tests and vital signs (94 labs, 15 vitals). 14 lab tests and vital signs had more than 90% missing values in the controls, and these were removed from both the cases and controls, bringing the total number of lab tests and vital signs to 95 (82 labs, 13 vitals). The distribution of lab tests and vital signs per control patient was approximately normal (Fig. 1c). Separate experiments were conducted where sparse lab tests and vital signs were imputed using the mean or the median. Histories of HIV, hepatitis C, and sickle cell disease were considered for case-control matching. As diagnoses of HIV and hepatitis C may happen after a patient was diagnosed with substance dependence, we labeled a patient as having HIV or hepatitis C if the diagnosis was made any time between birth and one year after diagnosis of substance dependence. Age, sex, and gender, along with status of HIV, hepatitis C, and sickle cell disease were used for case-control matching. For selecting an age for each control, the mean age was used from the patient’s respective lab tests and vital signs. Disease status was one-hot encoded, and gender was binary encoded. Nearest neighbor by Euclidean distance matching was applied with replacement to match 10 controls to each case. Accounting for matching with replacement, cases were matched to 43,243 unique controls. The data from these cases and matched controls was all recorded between 2000 and 2015. A schematic that illustrates the steps taken in constructing the case and control groups is provided (Additional file 1: Figure S1).

### Methods to compare cases and controls

Using the lab tests and vital signs from the cases and matched controls, median effect sizes were calculated for all 95 lab tests and vital signs. The value of the lab test or vital sign from each case is used to calculate an effect size, then for each lab test or vital sign, the median of these effect sizes is taken. Medians were calculated because they are more robust to outliers. Mean effect sizes were also calculated to check for consistency of their direction. Additionally, we examined the values of the lab tests and vital signs in both cases and controls during the 100 days prior to the diagnosis of substance dependence. For comparing these profiles to the controls, we examined the 100 days prior to the mean day of their lab tests and vital signs. Finally, diagnoses, prescriptions, and procedures in the five years preceding the first diagnosis of substance dependence were compared to those found in the age matched controls using odds ratios.

### Opioid prescriptions analysis

To examine opioid prescriptions in the MSMC-EHR, the percentages of patients with at least one or more opioid prescriptions were calculated along with the percentage of total opioid prescriptions. Additionally, the distribution of opioid prescriptions by patient was examined, and a Wilcoxon rank-sum test was applied to quantify the difference between the number of opioid prescriptions given to patients with an opioid dependence prior to the substance abuse diagnosis, and the number of opioid prescriptions given to patients who have at least one opioid prescription, but no history of opioid dependence.

### Classification of patients by substance dependence status

A Random Forest classifier was implemented with Scikit-learn [22] with 100 estimators, a Gini criterion, and a random state of 42. Cases and matched controls were iteratively classified using a bootstrapping procedure. 100 bootstraps of equal size to the case population were sampled from the matched controls, and 10-fold cross-validation was applied on each bootstrap. Area under the receiver operating characteristic curve (AUROC) was calculated as one way to assess classifier performance [23]. Gini importance was measured for each lab test and vital sign to assess the contribution of each feature. The 10 lab tests and vital signs (features) with the highest Gini importance were tested, and AUROCs were calculated. 10 random sets of 10 features were tested to determine baseline performance using random lab tests and vital signs. Finally, a dummy classifier making predictions by randomly picking from the population was employed to establish a performance baseline. F1 scores were calculated for all precision-recall combinations along the precision-recall curve, and confusion matrices were calculated using the threshold corresponding to the highest F1 score.

In the experiments using imputation by the mean or the median, classification performance was measured for including all patients, only those with no less than 17 lab tests and vital signs, and only those with less than 17 lab tests and vital signs. Additionally, we ran a test case with patients that had ICD-9 code families in the range of 291–293 but did not have ICD-9 codes in the 304.* family. These 291–293 ICD codes denote alcohol- and drug-induced mental disorders and withdrawal. Because the data in these analyses had higher dimensionality, i.e., more lab tests and vital signs due to the retention of all patients, only 10 bootstraps of equal size were sampled from the matched controls, and 10-fold cross-validation was conducted on each bootstrap.

The lab tests and vital signs during the 20 days prior to the first diagnosis of substance dependence, as well as 10 days before and 10 days after the first diagnosis, were used as the features to train the main set of classifiers. However, other classifiers were developed using only the diagnoses, prescriptions, and procedures during the 5 years prior to the first diagnosis of substance dependence. Furthermore, rather than predicting substance dependence status, we also constructed models to predict non-medical opioid poisoning events, i.e. overdose, denoted by ICD-9 codes 965.0, 965.00, 965.01, 965.02, 965.09, E850.0, E850.1, E850.2, using lab tests and vital signs during the 6 months prior to the event.

## Results

### Descriptive statistics of the case population

**Fig. 2:**
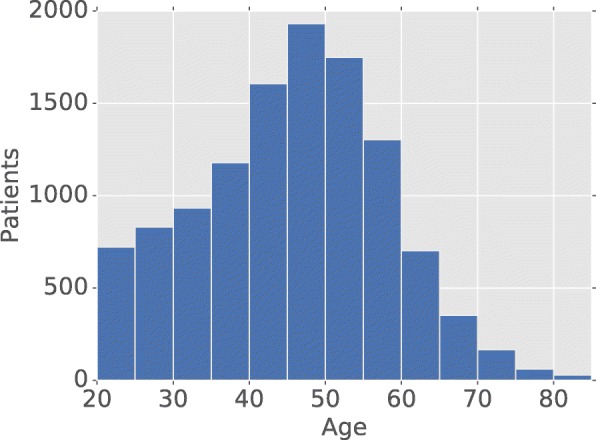
Distribution of ages for 11,573 cases given their first substance dependence diagnoses at 20 years of age or older

From the 12,112 patient records within the MSMC-EHR that had at least one substance dependence diagnosis, 64.12% were males (7745) and 35.84% were females (4329). Out of these we retained 11,573 cases whose first diagnosis of substance dependence was made at 20 years of age or later. The mean age of these patients at their first substance dependence diagnosis was 45.6 years, with the youngest patient being 20 years old, and the oldest 89.4 (Fig. 2). 9528 of these patients had 1,525,293 recorded lab tests and vital signs measurements during the period of 10 days before and 10 days after their diagnosis of substance dependence. 9518 cases remained after outlier removal. The final case population was obtained after removing patients with less than 17 lab tests and vital signs, leaving 7797 patients. The case population consists of 65.4% males (5103), and 34.6% females (2694). The breakdown of ICD 304.* sub-diagnoses by drug is 53.5% opioids (4168), 19.98% cocaine (1558), 9.4% cannabis (736), 7.0% combo without opioids (547), 4.3% unspecified (337), 3.8% sedatives (295), 2.2, 1.2% amphetamines (94), and 0.8% other (59).

### Opioid prescriptions in the Mount Sinai EHR

**Fig. 3:**
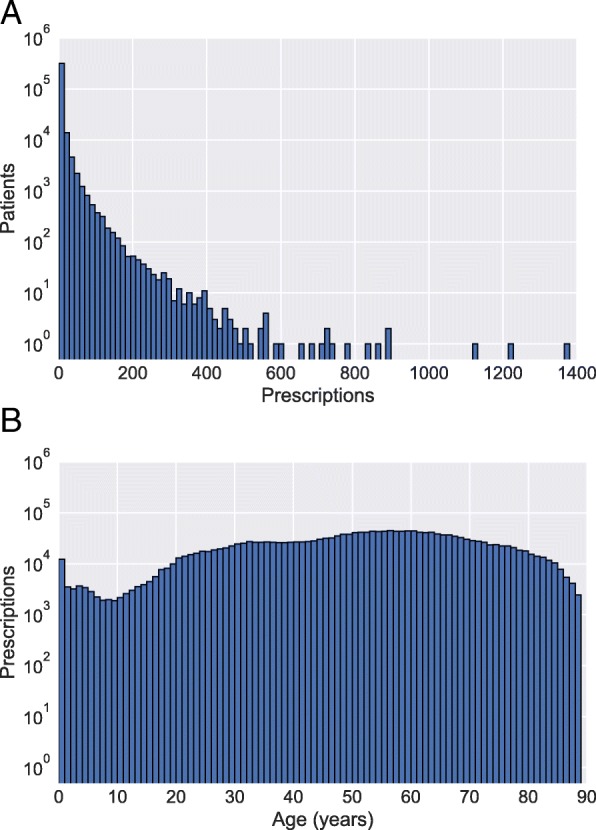
Histograms of (**a**) opioid prescriptions per patient, and (**b**) prescriptions per age in years

Out of the 880,605 patients that have at least one prescription in the MSMC-EHR, 356,734 patients (40.51%) had at least one opioid prescription. Out of 45,392,334 total prescriptions, 2,029,008 prescriptions (4.47%) were for opioids. While 356,734 patients had at least one opioid prescription, 214,757 had at least two, 155,120 had at least three, 118,208 had at least four, 42,515 had at least five, and 5312 had at least fifty (Fig. 3). Prescriptions showed an approximate uniform distribution across ages 20–80, with a slight increase for infants, possibly due to newborns born to substance dependent mothers (Fig. 3). Total prescriptions in the EHR steadily increased from the year 2000. For patients with an opioid dependence diagnosis, the average number of days between their first opioid prescription and first diagnosis of opioid dependence was 64 days. The median was one day, likely due to patients that are prescribed methadone for the treatment of their previously existing opioid dependence. Patients diagnosed with an opioid use disorder had significantly more opioid prescriptions in the EHR. An average of 26.7 opioid prescriptions were observed for patients with opioid use disorder diagnosis, and an average of 5.27 for patients without opioid use disorder diagnosis (*p* = 9.14E-208, Wilcoxon Rank Sum test). In terms of cases and their matched control populations, similar percentages had a prior non-methadone opioid prescription (cases: 24.57%, controls: 24.69%). However, for the patients that had a prior opioid prescription, the case patients had a mean of 13.07 prescriptions, and the controls had a mean of 4.25 prescriptions.

### Quantifying differences between the case and control groups

Effect size is a common method to measure differences between measured variables in case and control groups. Lab tests and vital signs with the highest median effect sizes were blood gases (pO2, O2 SAT, pCO2, CO2) and white blood cell (WBC) markers (lymphocytes, basophils) (Table 1). This can be explained by prior observations that indicated that respiratory disturbances are related to pain and pain scores [24, 25]. It is also possible that if patients use narcotics, their respiratory rate is suppressed, and this causes their pCO2 to rise. WBC counts have been noted to change in the short term in response to stressors such as surgery and trauma [26, 27]. Looking at the 100 days prior to diagnosis of substance dependence, pain score ratings are significantly elevated in the cases compared to controls at ~ 80 days prior to diagnosis (Additional file 2: Figure S2). This prior elevation in pain scores may indicate the typical time from an initial opioid prescription to the point of substance dependence diagnosis. However, it is established that progression from an opioid prescription to a diagnosis of dependence generally takes many months to years. It is alternatively likely, that a high early pain score suggests opioid tolerance, and a diminished threshold for pain.

**Table 1:** Top 10 lab tests and vital signs by median effect size

Lab Test/Vital Sign	Median Effect Size	Mean Effect Size
Absolute lymphocyte count	1.103	1.132
Oxygen saturation	1.1	1.289
Lymphocytes percentage	1.1	1.147
Partial pressure of oxygen	1.092	1.208
Estimated glomerular filtration rate	1.056	1.276
Total carbon dioxide level	1.048	1.067
Platelet count	1.045	1.081
Carbon dioxide pressure	1.044	1.072
Alkaline phosphatase	1.036	1.098
Aspartate aminotransferase	1.035	1.111

### Machine learning classifier to predict opioid dependence

Lab tests and vital signs from the cases and matched controls were used to train various Random Forest classifiers. A bootstrapping method was used to match different sets of controls to equal size of the case population. The initial set of *n* = 7797 case patients was achieved by the filtering steps described in the methods. Stratified 10-fold cross-validation was implemented to evaluate the performance of the classifiers.

### Classifiers that use only labs and vitals dense data without imputation

**Fig. 4:**
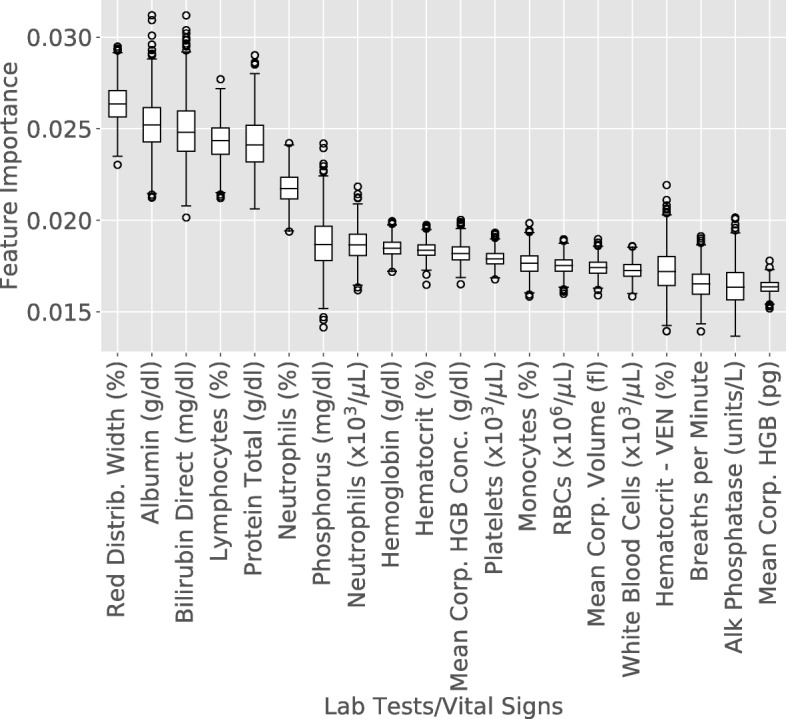
Gini importance values for the top 20 features by mean Gini importance

The initial Random Forest classifier achieved, across 100 bootstraps, each with 10 folds, AUROCs ranging from 0.813–0.875, with a mean of 0.846 (Additional file 3: Figure S3A). To select the optimal probability threshold for making a binarized prediction, F1 scores were calculated across all possible threshold values. The probability threshold (0.42) yielded the highest F1 score (0.776) and was used to calculate confusion matrices. The confusion matrix shows that the classifier correctly labeled 67.9% of the controls and 83.8% of the cases (Additional file 3: Figure S3B). When only using the lab tests and vital signs with the top 10 highest Gini important features, AUROCs dropped to a range from 0.72–0.796, with a mean of 0.76 (Additional file 3: Figure S3C). The probability threshold (0.38) that yielded the highest F1 score (0.722) was used to calculate confusion matrices showing the classifier correctly labeled 51.4% of controls and 84% of cases (Additional file 3: Figure S3D). Using 10 random sets of 10 features, AUROCs ranged from 0.587–0.789, with a mean of 0.72 (Additional file 4: Figure S4A). The probability thresholds across the 10 sets of features ranged from 0.16–0.36 that yielded the highest F1 scores in the range of 0.669–0.715, were used to calculate confusion matrices showing for each of the 10 sets that showed the classifiers correctly labeled 16.1–52.1% of controls and 82.2–92.4% of cases (Additional file 4: Figure S4B). Additionally, we tested the performance of random predictions from a dummy classifier to establish a baseline classification performance not using any features. AUROCs for all 10 folds ranged from 0.457–0.54, with a mean of 0.5 (Additional file 4: Figure S4C), and the confusion matrix showed the classifier correctly labeled 50% of controls and 50% of cases (Additional file 4: Figure S4D) as expected. The average precision (AP) scores for the classifiers were as follows: 0.843 (all features), 0.744 (top 10 features by Gini), 0.499 (dummy), and the range for the 10 sets of 10 random features was 0.615–0.751. Overall this analysis suggests that by using all features, classification improves. The features with the highest mean Gini importance across the 10 folds when using all features are related to white blood cells (lymphocytes, neutrophils), blood-specific measures RCDW, hematocrit, hemoglobin, bilirubin), and protein (total protein, albumin) (Table 2). These are consistent with boxplots of the Gini importance for the top 20 features (Fig. 4) and the raw values for the top nine features (Additional file 5: Figure S5). Additionally, we tested the classification performance using the top 10 lab tests and vital signs by median effect size. These lab tests and vital signs were sparser across patients, and hence performed significantly worse than the top 10 features by Gini importance and *p*-value. The mean AUROC was 0.619, with the confusion matrix showing correct identification of 11.6% of controls and 95.4% of cases.

**Table 2:** Top 10 features by Gini importance

Lab Test/Vital Sign	Mean Gini	SD Gini	Case Mean	Control Mean	Case SD	Control SD
Red blood cell distribution width	0.026	0.001	14.620	14.1	1.687	1.592
Albumin testing g/dL	0.025	0.001	3.793	3.99	0.753	0.749
Total bilirubin mg/dL	0.025	0.002	0.191	0.19	0.115	0.111
Lymphocytes percentage	0.024	0.001	25.509	22.236	10.253	10.488
Total protein g/dL	0.024	0.001	7.181	7.078	0.855	0.832
Neutrophils percentage	0.022	0.001	63.032	67.512	12.542	12.707
Phosphorus mg/dL	0.019	0.001	3.608	3.482	0.729	0.742
Absolute neutrophil count	0.019	0.001	4.826	5.566	2.396	2.674
Hemoglobin g/dL	0.018	0	12.545	12.886	2.021	2.031
Hematocrit test	0.018	0	37.164	38.05	5.851	5.892

### Classifiers that use imputed data with all labs and vitals

Because a significant number of lab tests, vital signs, and patients were discarded due to sparsity, an alternative approach is to impute the missing values with expected values. Using the imputation strategies of substituting missing values with the mean or the median yielded similar results. Without imputation, the AUROCs ranged from 0.832–0.87, with a mean AUROC of 0.856 (Additional file 6: Figure S6A). The confusion matrix showed that the classifier correctly identified 74.5% of controls and 81% of cases (Additional file 6: Figure S6B). Imputing by the mean, the AUROCs ranged from 0.822–0.87, with a mean AUROC of 0.847 (Additional file 6: Figure S6C). The confusion matrix showed that the classifier correctly identified 72.7% of controls and 80.9% of cases (Additional file 6: Figure S6D). Imputing by the median, the AUROCs ranged from 0.824–0.867, with a mean of 0.844 (Additional file 6: Figure S6E). The confusion matrix showed that the classifier correctly identified 72.1% of controls and 81% of cases (Additional file 6: Figure S6F).

### Classifiers that use only dense labs and vitals data with imputation

**Fig. 5:**
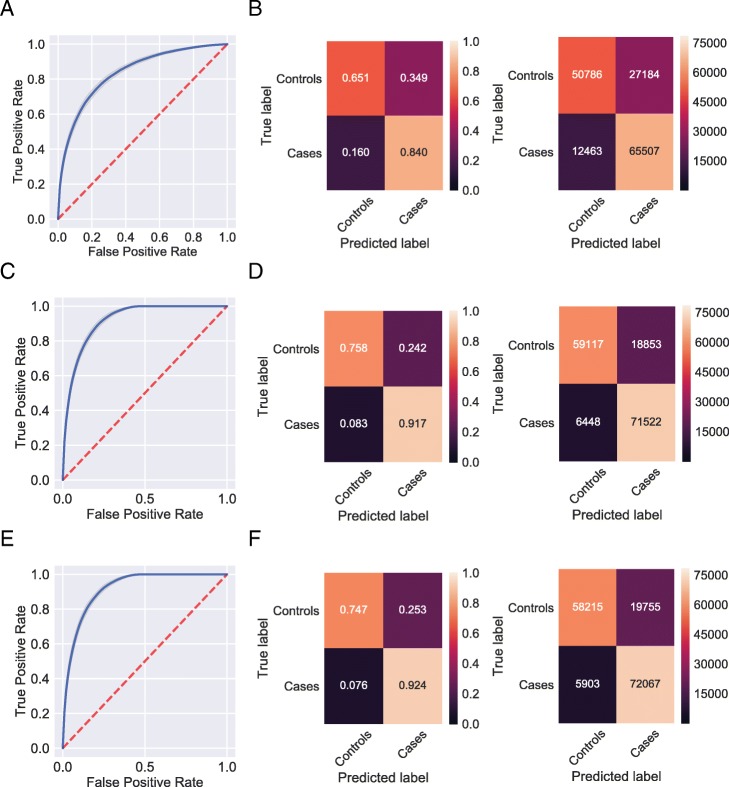
Receiver operating characteristic curves, normalized, and non-normalized confusion matrices for classifiers using no imputation (**a**, **b**), imputation by the mean (**c**, **d**), and imputation by the median (**e**, **f**), retaining only patients with more than 17 labs and vitals

The imputation strategies increased the AUROC when limiting to the original set of non-sparse patients to patients with at least 17 or more lab tests and vital signs. When retaining only patients with non-sparse data and using no imputation, AUROCs ranged from 0.805–0.858, with a mean of 0.835 (Fig. 5a). The confusion matrix showed that the classifier correctly identified 65.1% of controls and 84% of cases (Fig. 5b). Retaining non-sparse patients and imputing by the mean, AUROCs improved to 0.902–0.931 with a mean of 0.918 (Fig. 5c). The confusion matrix showed that the classifier correctly identified 75.8% of controls and 91.7% of cases (Fig. 5d). Retaining non-sparse patients and imputing by the median, AUROCs improved to 0.899–0.933, with a mean of 0.917 (Fig. 5e). The confusion matrix showed the classifier correctly identified 74.7% of controls and 92.4% of cases (Fig. 5f). Hence, by imputing patients with dense data, we achieved the maximally improved quality predictions.

### Patients with drug or alcohol induced mental disorders as a test case

In constructing our control sample, we excluded patients with a diagnosis of a drug- or alcohol-induced mental disorders, specifically, ICD-9 codes in the range of 291–293. Examining these patients as a potential test cases, there were 6573 patients who had these ICD-9 codes, but only 1466 of these patients had the 291–293 ICD-9 codes without additional diagnosis in the 304.* family. The classifier predicted that 57.6% of these patients belonged to the case group, compared to 21.3% of an equally-sized set of matched controls, suggesting that patients with drug- or alcohol-induced mental disorders are much more likely to also misuse opioids and develop dependence.

### Classifiers that use lab test and vital signs from 20 day prior to initial diagnosis

So far, all classifiers described used vital signs and lab test from 10 day prior to initial diagnosis of substance dependence and 10 day post this diagnosis. Next, we modified the cases dataset to include only lab tests and vital signs during the 20 days prior to the initial diagnosis of substance dependence. We did this to assess whether the machine learning approach can operate in a practical clinical setting before diagnosis of substance dependence is detected and reported. Without imputation, AUROCs ranged from 0.791–0.857, with a mean of 0.833 (Additional file 7: Figure S7A). The confusion matrix showed that the classifier correctly identified 64.4% of controls and 84.5% of cases (Additional file 7: Figure S7B). Imputing by the mean, AUROCs ranged from 0.787–0.85, with a mean of 0.823 (Additional file 7: Figure S7C). The confusion matrix showed that the classifier correctly identified 63.8% of controls and 83.1% of cases (Additional file 7: Figure S7D). Imputing by the median, AUROCs ranged from 0.781–0.849, with a mean of 0.82 (Additional file 7: Figure S7E). The confusion matrix showed that the classifier correctly identified 66.4% of controls and 81.1% of cases (Additional file 7: Figure S7F). For these classifiers using lab tests and vital signs from 20 days prior to diagnosis of substance dependence, the AP scores were as follows: 0.829 (no imputation, Additional file 8: Figure S8A), 0.821 (mean imputation, Additional file 8: Figure S8B), and 0.818 (median imputation, Additional file 8: Figure S8C). Hence, we can retain comparable high quality predictions by shifting the window of 20 days to those days before initial diagnosis.

### Classifiers that use diagnoses, prescriptions, and procedures

In addition to predicting substance dependence status from lab tests and vital signs, we also tested whether substance dependence status could be predicted only from 5-year clinical history of diagnoses, prescriptions, and procedures. Total number of diagnoses, prescriptions, and procedures from the 5 years before the first diagnosis of substance dependence were classified, with and without imputation. Without imputation, AUROCs ranged from 0.838–0.889, with a mean of 0.863 (Additional file 9: Figure S9A). The confusion matrix showed the classifier correctly identified 75.2% of controls and 81.8% of cases (Additional file 9: Figure S9B). Ranking all diagnoses, prescriptions, and procedures by Gini importance, the top 10 features were: methadone prescription, major depression diagnosis, trazodone prescription (used to treat major depression), interview/evaluation procedure, nicotine prescription, sodium chloride prescription, thiamine prescription, HIV diagnosis, lorazepam prescription, and personal history of allergy to penicillin diagnosis. Imputing by the mean, AUROCs ranged from 0.827–0.875, with a mean of 0.853 (Additional file 9: Figure S9C). The confusion matrix showed that the classifier correctly identified 72% of controls and 82.4% of cases (Additional file 9: Figure S9D). Imputing by the median, AUROCs ranged from 0.796–0.858, with a mean of 0.821 (Additional file 9: Figure S9E). The confusion matrix showed the classifier correctly identified 72.1% of controls and 75.4% of cases (Additional file 9: Figure S9F). For these classifiers, using the 5-year clinical history of diagnoses, prescriptions, and procedures prior to diagnosis of substance dependence, the AP scores were as follows: 0.865 (no imputation, Additional file 10: Figure S10A), 0.849 (mean imputation, Additional file 10: Figure S10B), and 0.829 (median imputation, Additional file 10: Figure S10C). Hence, we conclude that this strategy is also highly predictive. The most important features are consistent with the features described below when clinical phenotyping was applied to the original classifiers that utilized vital signs and lab tests.

### Classifiers that predict overdose

Aside from predicting substance dependence status, we tested whether the diagnosis of a non-medical opioid poisoning, an overdose, could be predicted from lab tests and vital signs from data collected 6 months prior to the overdose event, with and without imputation. Lab tests and vital signs from the 6 months before the diagnosis of a non-medical opioid poisoning were classified, with and without imputation. Because these cases and control populations were small (477 cases, 4527 matched controls), the results showed more variability. Without imputation, AUROCs ranged from 0.694–0.922, with a mean of 0.822 (Additional file 11: Figure S11A). The confusion matrix showed the classifier correctly identified 67.2% of controls and 80.7% of cases (Additional file 11: Figure S11B). Imputing by the mean, AUROCs ranged from 0.69–0.951, with a mean of 0.815 (Additional file 11: Figure S11C). The confusion matrix showed that the classifier correctly identified 69.2% of controls and 79.3% of cases (Additional file 11: Figure S11D). Imputing by the median, AUROCs ranged from 0.665–0.933, with a mean of 0.811 (Additional file 11: Figure S11E). The confusion matrix showed that the classifier correctly identified 72.1% of controls and 77.5% of cases (Additional file 11: Figure S11F). Overall, these results suggest that non-medical opioid poisoning is somewhat predictive with prior knowledge about vital signs and lab tests. It is expected that with more cases, prediction quality will improve.

### Clinical phenotyping of cases based on diagnoses, prescriptions, and procedures

**Fig. 6:**
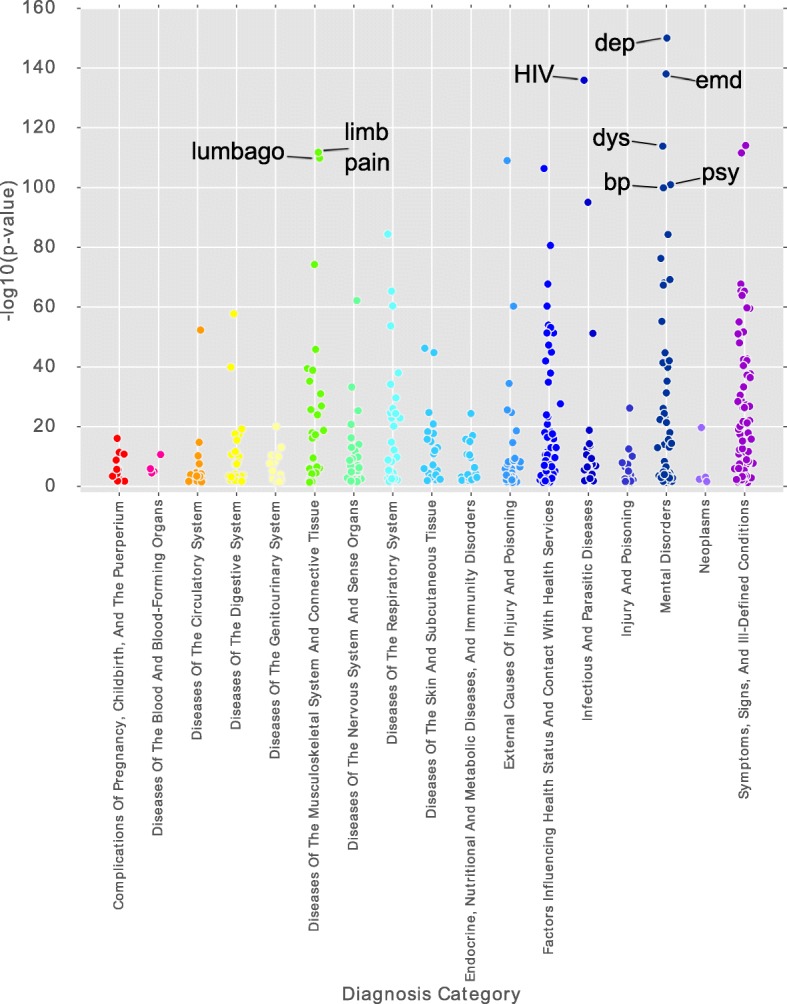
Scatter plot of 483 diagnoses with statistically significant over- or underrepresentation in the cases compared to the controls measured using the Fisher Exact test. Each point is a diagnosis that was statistically significant. 31 diagnoses have odds ratios of less than 1

The most differentially represented diagnoses during the 5 years prior to the diagnosis of substance abuse in the cases were mostly psychiatric, including depression, episodic mood disorder, dysthymic disorder, bipolar disorder, and unspecified psychosis (Fig. 6, Table 3). This supports the observation that patients with substance abuse are more likely to have psychiatric conditions than non-substance abusers, and these conditions may predispose patient toward substance abuse. Human immunodeficiency virus (HIV) and hepatitis C were overrepresented in the cases. It is known that globally, intravenous drug users are 28 times more likely to contract HIV than the rest of the adult population [28]. Intravenous drug use is also responsible for ~ 90% of new hepatitis C infections [29].

**Table 3:** Top 10 differentially represented diagnoses during the 5 years prior to diagnosis of substance abuse (ranked by odds ratio)

Diagnosis	Odds ratio	*p*-value (Bonferroni-corrected)
Unspecified episodic mood disorder	11.779	1.03E-138
Dysthymic disorder	6.209	1.48E-114
Depressive disorder, not elsewhere classified	6.081	0
Personal history of noncompliance with medical treatment, presenting hazards to health	5.933	4.29E-107
Other unknown and unspecified cause of morbidity and mortality	4.896	2.69E-112
Accidents occurring in unspecified place	4.845	9.87E-110
Pain in limb	4.565	1.66E-112
Cough	4.54	9.00E-115
Lumbago	4.301	1.54E-110
Human immunodeficiency virus [HIV] disease	3.467	1.28E-136

Medical non-adherence, a condition where patients do not follow therapeutic recommendations, is overrepresented in the cases. This finding may support a socioeconomic difficulty in adhering to medical advice, or general apathy to medical treatment, or a refusal to take alternative medications that are not opioids, or a refusal for any psychiatric treatment. Lumbago, an older term for low back pain, is also overrepresented in the cases. Patients with lumbago are often treated with opioids, and may become addicted; or conversely, opioid users with correspondingly lower thresholds for pain may present to clinics or emergency departments with lumbago. Other pain-related diagnoses are also overrepresented in the cases. These include limb pain (OR = 4.56, *p* = 1.66E-112), backache (OR = 4.04, *p* = 5.82E-75), abdominal pain (OR = 2.68, *p* = 5.23E-66), chronic pain (OR = 6.47, *p* = 6.32E-63), chest pain (OR = 1.95, *p* = 3.49E-43), and others. Diagnoses underrepresented in the cases include those related with pregnancy, such as “supervision of other normal pregnancy,” “outcome of delivery, single liveborn,” and “post term pregnancy, delivered, with or without mention of antepartum condition.” This is related to the suggestion that pregnant patients are among the least likely to seek care from multiple institutions, while HIV and chronic pain patients are among the most likely to seek care from multiple institutions [30].

Among prescriptions that are differentially represented during the 5 years prior to the first diagnosis of substance abuse in the cases are nicotine patches. It was previously reported that 85–98% of patients undergoing methadone maintenance treatment consume tobacco [31]. Other differentially represented medications among cases include the psychoactive medication trazodone, as well as the “banana bag” cocktail of thiamine, folic acid, and multivitamins with sodium chloride, given to malnourished alcohol users [32]. Important to note, these are not prescriptions but rather inpatient orders. Other prescriptions are for methadone and lorazepam. Lorazepam is an anxiety medication that is also used for alcohol withdrawal symptoms (Table 4). Prescriptions underrepresented in the cases included cefazolin, an antibiotic; ondansetron, a 5HT-3 antagonist used as an anti-emetic; and midazolam, a short-acting sedative. Midazolam is a benzo, just like lorazepam, but shorter-acting. Lorazepam is given to alcohol withdrawal patients and agitated patients, while midazolam is given for procedural sedation such as shoulder dislocation, or lumbar puncture. Medical procedures that are differentially represented during the 5 years prior to the diagnosis of substance abuse in the cases are various forms of evaluations and interviews, in agreement with the overrepresentation of psychiatric diagnoses (Table 5).

**Table 4:** Top 10 differentially represented prescriptions during the 5 years prior to diagnosis of substance abuse (ranked by odds ratio)

Prescription	Odds ratio	*p*-value (Bonferroni-corrected)
Methadone	45.956	0
Nicotine	26.239	0
Thiamine	12.861	1.58E-277
quetiapine	11.553	3.27E-241
Trazodone	9.863	0
Clonazepam	8.38	7.21E-165
Haloperidol	6.82	8.28E-178
Folic Acid	5.28	5.03E-202
Lorazepam	4.745	2.02E-231
Ibuprofen	4.64	2.58E-202

**Table 5:** Top 10 differentially represented procedures during the 5 years prior to diagnosis of substance abuse (ranked by odds ratio)

Procedure	Odds ratio	p-value (Bonferroni-corrected)
Other group therapy	19.8	1.23E-69
Interview & Evaluation NEC	11.578	4.06E-42
Psychiatric Mental Determination	10.597	1.24E-29
Exploratory verbal psychotherapy	10.371	2.41E-57
Brief interview & evaluation	6.224	9.00E-201
Limited interview/evaluation	5.804	2.71E-279
Interview & evaluation NOS	5.745	6.16E-102
Comprehensive interview/evaluation	5.065	1.71E-147
Other counselling	4.3	3.93E-36
Other fetal monitoring	0.218	1.14E-30

## Discussion

Using lab tests and vital signs proximal to the diagnosis date of substance dependence as input, we tested the ability of a Random Forest machine learning classifier to predict whether a patient will be diagnosed with substance dependence. Using a baseline of 50/50 chance to diagnose a patient as substance dependent, the best classifier performed well above chance. The best classifier correctly predicted whether a patient is not substance-dependent ~ 76% of the times, and whether a patient is a substance-dependent ~ 92% of the times. While these results are promising, there is room for improvement before a clinical implementation. The measurements that distinguished substance-dependent patients from non-substance dependent patients, as determined by effect size, Gini importance, or by the Wilcoxon rank-sum test, were mostly related to white blood cells, protein, blood gases, blood volume and blood cell width. The relationships between these lab tests and vital signs in the context of substance dependence diagnosis can be explained. It is encouraging that the laboratory results and vital signs identified by the classifier have well-known relationships to pain syndromes and opioid use. Respiratory rate, for instance, has been shown to be elevated in many painful conditions, and decreased in opioid overdose. Respiratory rate will directly affect blood gases. White blood cell counts have also been shown to fluctuate in response to trauma and surgery, with a decline in lymphocytes and an increase in polymorphonuclear leukocytes (PMNs). Compared to the substance-dependent cases, our control cohort showed the same pattern as prior studies of trauma patients. In addition to classification using clinical measures, we attempted to classify patients with, as well as examined the prevalence of, diagnoses, prescriptions, and procedures in the case and control populations during the five years before diagnosis of substance dependence. The diagnoses most overrepresented were psychiatric, supporting the close association between substance abuse and psychiatric comorbidities as reported before [7–10]. Agreeing with this, the medical procedures most overrepresented in the cases were various types of psychiatric evaluations and interviews. The prescriptions most overrepresented in the cases were related to opioid treatment and malnourishment, as many drug abusers arrive at the hospital in a malnourished state. Examining all opioid prescriptions in the MSMC-EHR, opioids were prescribed to a large portion of patients, and patients diagnosed with an opioid use disorder had significantly more opioid prescriptions than patients who were given few opioid prescriptions. Future work may include other features for predicting substance dependence status. These can be combined with the clinical features we already used here. Additionally, other machine learning methods such as deep learning may perform better than the Random Forest classifier we employed. The case and control populations could also be made larger by integrating other EHR systems. It is possible that results will vary when examining distinct populations across hospitals in different cities and countries. The current study is focused on patients with diagnoses in the 304 family (drug dependence), but there are other ICD-9 families related to drug abuse. The 305 family, which denotes non-dependent substance abuse, was commonly used for patients with alcohol and tobacco use disorders. For this reason, we focused on the 304 family of ICD-9 diagnoses. Finally, future studies can examine gene variants that are enriched in the cases compared to the controls. Such analysis can identify genetics variants that may influence propensity for drug abuse and at the same time point further to mechanisms of action. The machine learning classifiers developed here can increase the size of the case population to improve the statistical power needed to identify true variants.

## Conclusions

Through analyzing of the health records of hundreds of thousands individuals in the MSMC-EHR with a machine learning framework, we furthered characterized opioid dependent patients using physiological measurements. We found that opioid dependent patients have significantly higher WBC and respiratory disturbances. Opioid dependent patients are also commonly malnourished which is characterized by low RCDW and blood albumin compared to controls. Clinical phenotyping analysis discovered that opioid dependent patients are more likely to suffer from psychiatric disorders and manifest pain-related symptoms. The predictive model may hold utility for identifying patients at risk of developing dependence, risk of overdose, and opioid-seeking patients that report other symptoms in their visits to the emergency room. It should be noted that marking a patient with an opioid dependency ICD code, which is commonly used for insurance purposes, is often inaccurate and inconclusive. Hence, we recommend that the results from our study should be considered preliminary, and the quality of the real cases disputable. The study should be validated by other independent EHR systems and different computational approaches. Regardless, the multi-variate non-linear characteristic of the classifiers developed here, combine unique mixture of the values of many measured variables together to produce predictions not possible by looking at a single biomarker. The complex relationships between measured variables would be difficult to detect via an in-person clinical assessment alone. Hence, the predictive machine learning classifiers we developed can alert physicians about the potential of patients to have opioid dependency from routine lab tests and vital signs. However, there are still technical, administrative, and bureaucratic barriers for real implementation.

## Additional files

Additional file 1: **Figure S1.** Flowchart illustrating the steps of the creating the case and control populations. (PDF 131 kb)

Additional file 2: **Figure S2.** Pain score ratings for cases (purple) and controls (gray) during the 100 days prior to diagnosis of substance dependence. For the controls, the 100 days are from prior to their mean day of analyzed lab tests and vital signs. The lines represent a moving average. (PDF 104 kb)

Additional file 3: **Figure S3.** Receiver operating characteristic curves, normalized, and non-normalized confusion matrices for classifiers using all features (A, B), the top 10 by Gini importance (C, D), and the top 10 by *p*-value from the Wilcoxon rank-sum test (E, F). (PDF 419 kb)

Additional file 4: **Figure S4.** Receiver operating characteristic curves, normalized, and non-normalized confusion matrices for a classifier using 10 sets of 10 random features (1/10 are shown) (A, B), and a dummy classifier outputting random predictions (C, D). (PDF 44 kb)

Additional file 5: **Figure S5.** Raw values for cases and matched controls of the top 9 lab tests and vital signs by mean Gini importance. (PDF 174 kb)

Additional file 6: **Figure S6.** Receiver operating characteristic curves, normalized, and non-normalized confusion matrices for classifiers using no imputation (A, B), imputation by the mean (C, D), and imputation by the median (E, F). (PDF 602 kb)

Additional file 7: **Figure S7.** Receiver operating characteristic curves, normalized, and non-normalized confusion matrices for labs and vitals from the 20 days prior to substance dependence diagnosis, classified using no imputation (A, B), imputation by the mean (C, D), and imputation by the median (E, F). (PDF 1335 kb)

Additional file 8: **Figure S8.** Precision-recall curves for labs and vitals from the 20 days prior to substance dependence diagnosis, using classifiers with no imputation (A), imputation by the mean (B), imputation by the median (C). (PDF 199 kb)

Additional file 9: **Figure S9.** Receiver operating characteristic curves, normalized, and non-normalized confusion matrices for diagnoses, prescriptions, and procedures from the 5 years prior to substance dependence diagnosis, classified using no imputation (A, B), imputation by the mean (C, D), and imputation by the median (E, F). (PDF 596 kb)

Additional file 10: **Figure S10.** Precision-recall curves for classifiers using diagnoses, prescriptions, and procedures from the 5 years to substance dependence diagnosis, with no imputation (A), imputation by the mean (B), imputation by the median (C). (PDF 212 kb)

Additional file 11: **Figure S11.** Receiver operating characteristic curves, normalized, and non-normalized confusion matrices for lab tests and vital signs from the 6 months prior to opioid poisoning diagnosis, classified using no imputation (A, B), imputation by the mean (C, D), and imputation by the median (E, F). (PDF 597 kb)
