# Supplementary material for: Predicting opioid dependence from electronic health records with machine learning
Source: BioData Min. 2019 Jan 29;12:3. doi: 10.1186/s13040-019-0193-0 (PMC6352440; doi:10.1186/s13040-019-0193-0)
Supplement: Supplementary file 1 — Figure S1. Flowchart illustrating the steps of the creating the case and control populations. (PDF 131 kb) [file 13040_2019_193_MOESM1_ESM.pdf]

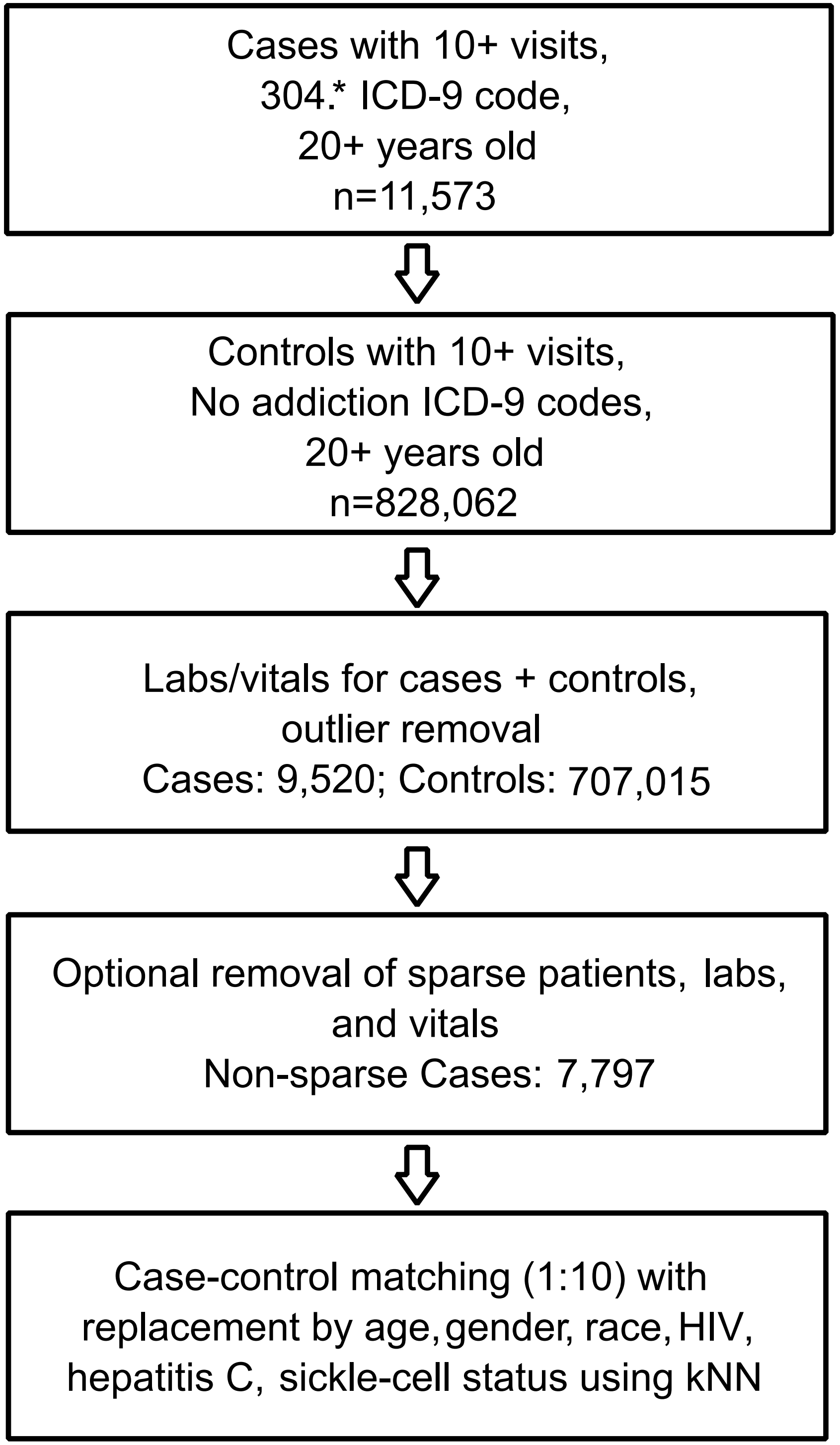
Cases with 10+ visits,
304.* ICD-9 code,
20+ years old
n=11,573
Controls with 10+ visits,
No addiction ICD-9 codes,
20+ years old
n=828,062
Labs/vitals for cases + controls,
outlier removal
Cases: 9,520; Controls: 707,015
Optional removal of sparse patients, labs,
and vitals
Non-sparse Cases: 7,797
Case-control matching (1:10) with
replacement by age, gender, race, HIV,
hepatitis C, sickle-cell status using kNN
