# Supplementary figures and images for: Predicting opioid dependence from electronic health records with machine learning
Source: BioData Min. 2019 Jan 29;12:3. doi: 10.1186/s13040-019-0193-0 (PMC6352440; doi:10.1186/s13040-019-0193-0)

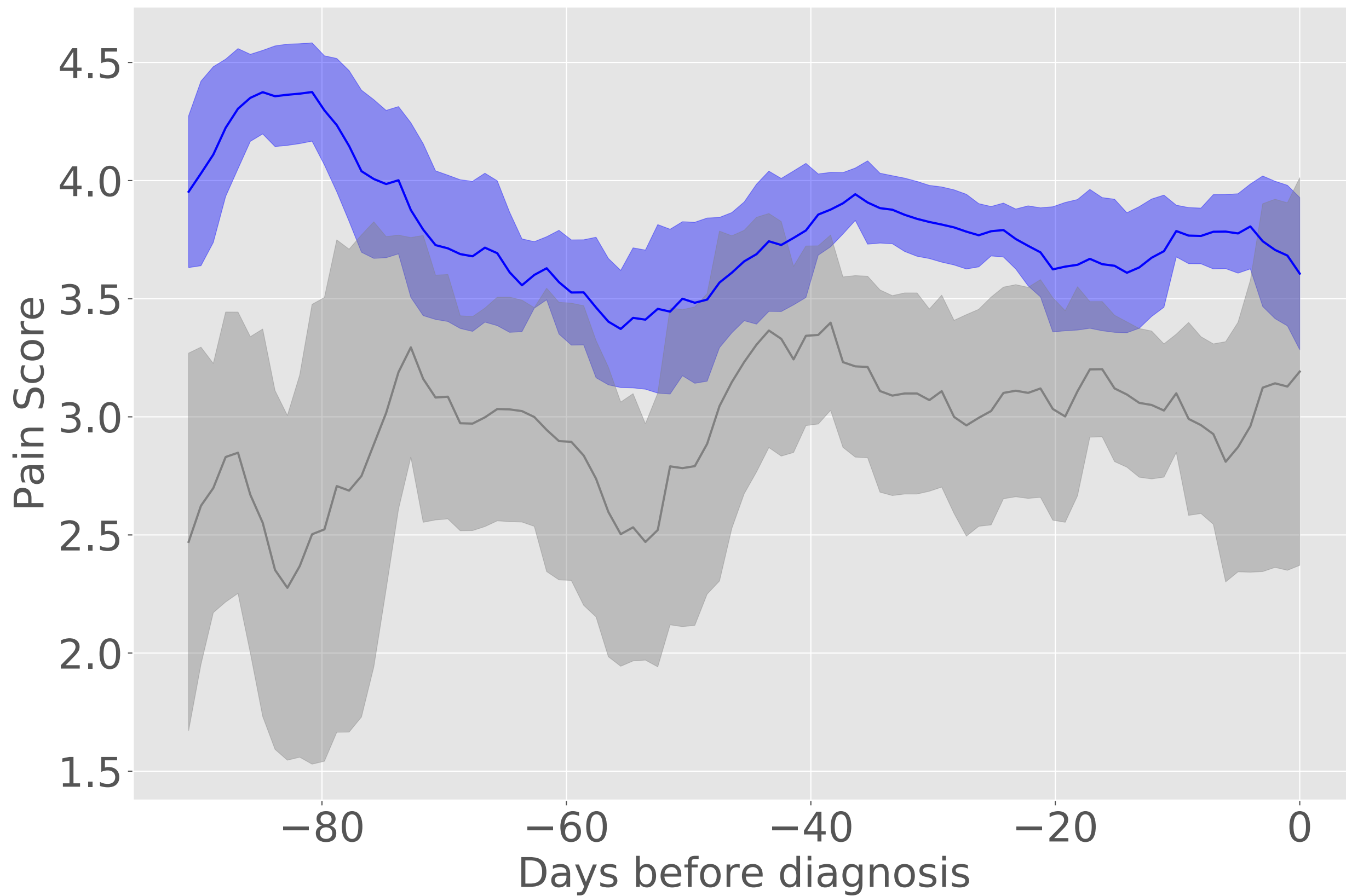

Supplement: Supplementary file 2 — Figure S2. Pain score ratings for cases (purple) and controls (gray) during the 100 days prior to diagnosis of substance dependence. For the controls, the 100 days are from prior to their mean day of analyzed lab tests and vital signs. The lines represent a moving average. (PDF 104 kb) [file 13040_2019_193_MOESM2_ESM.pdf]

A

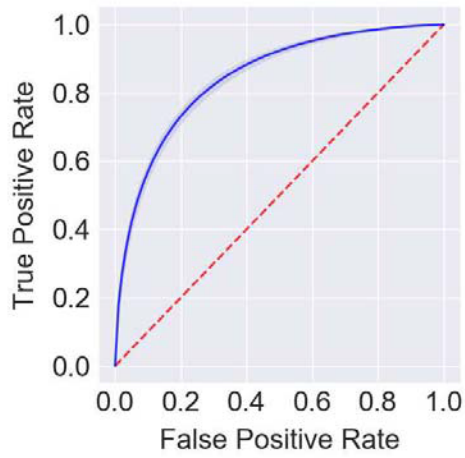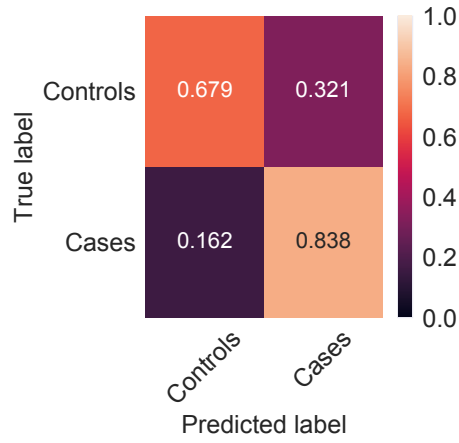

B

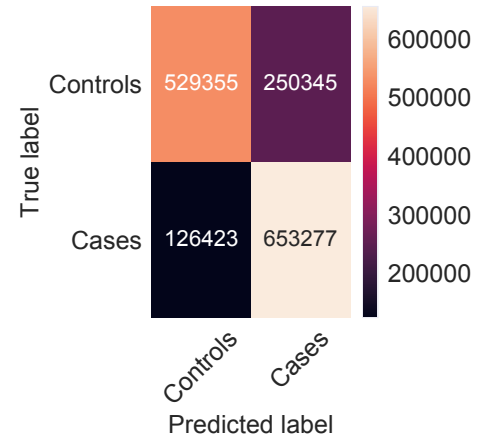

C

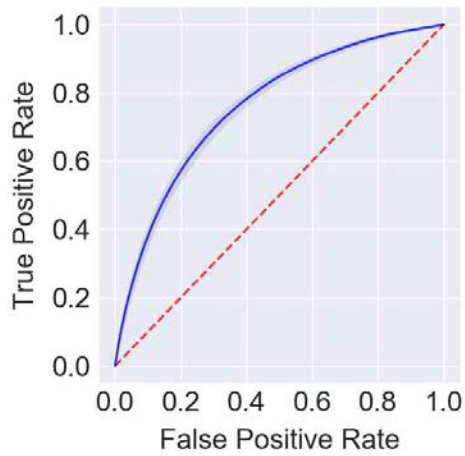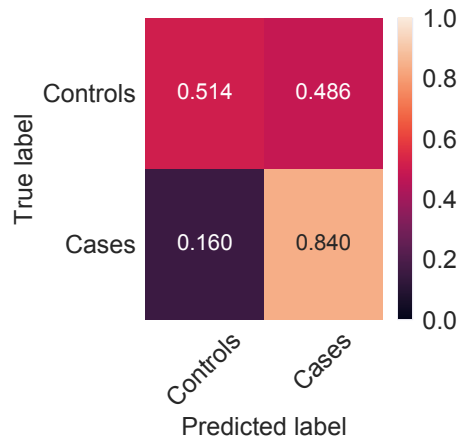

D

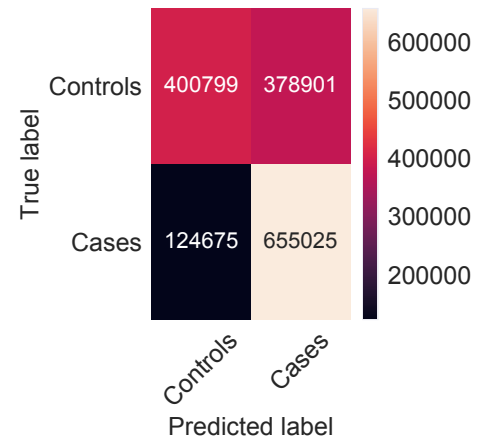

Supplement: Supplementary file 3 — Figure S3. Receiver operating characteristic curves, normalized, and non-normalized confusion matrices for classifiers using all features (A, B), the top 10 by Gini importance (C, D), and the top 10 by p-value from the Wilcoxon rank-sum test (E, F). (PDF 419 kb) [file 13040_2019_193_MOESM3_ESM.pdf]

A

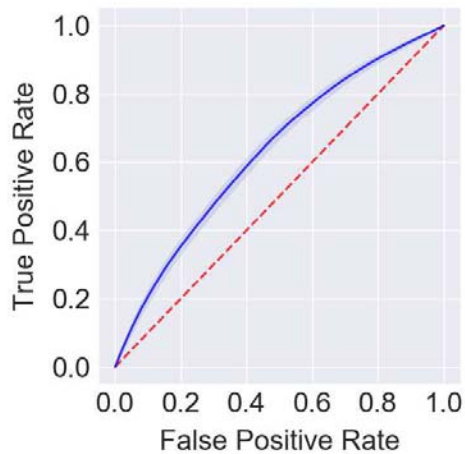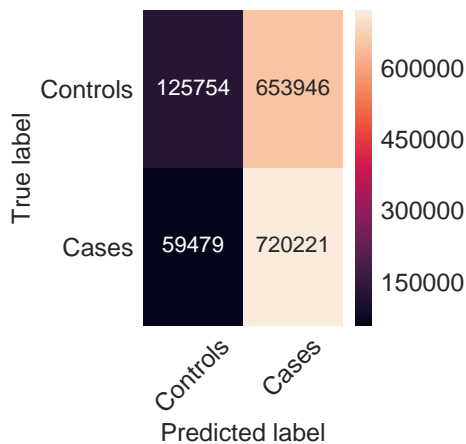

B

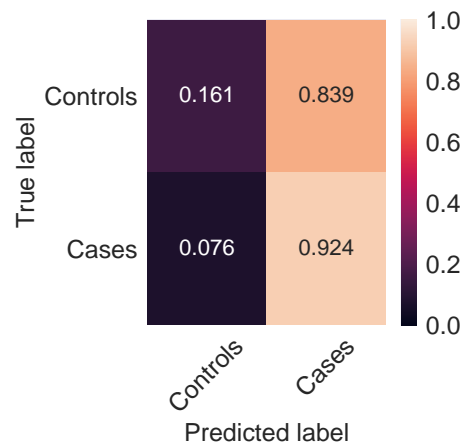

C

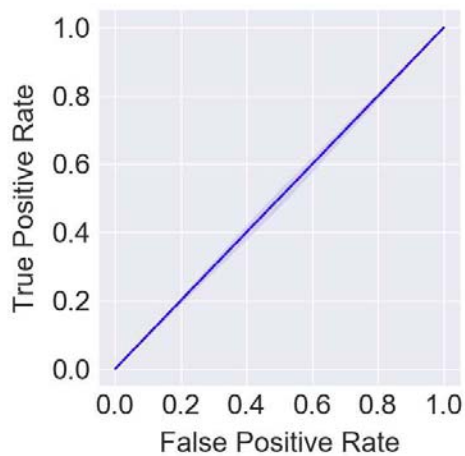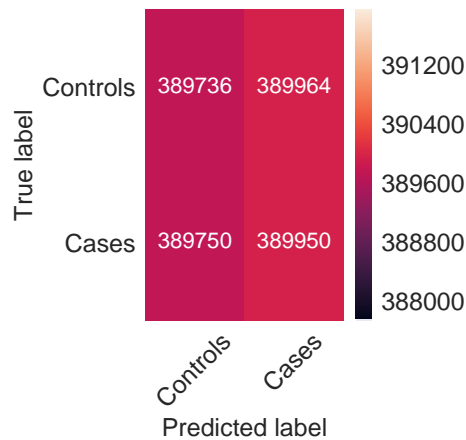

D

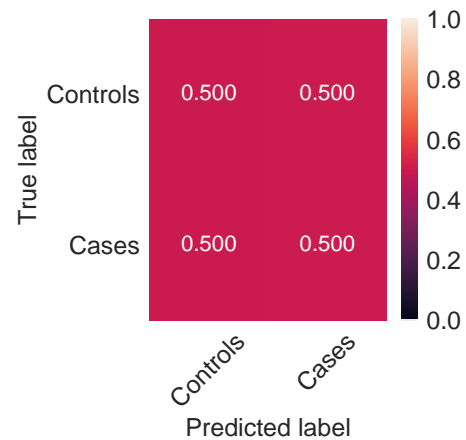

Supplement: Supplementary file 4 — Figure S4. Receiver operating characteristic curves, normalized, and non-normalized confusion matrices for a classifier using 10 sets of 10 random features (1/10 are shown) (A, B), and a dummy classifier outputting random predictions (C, D). (PDF 44 kb) [file 13040_2019_193_MOESM4_ESM.pdf]

A

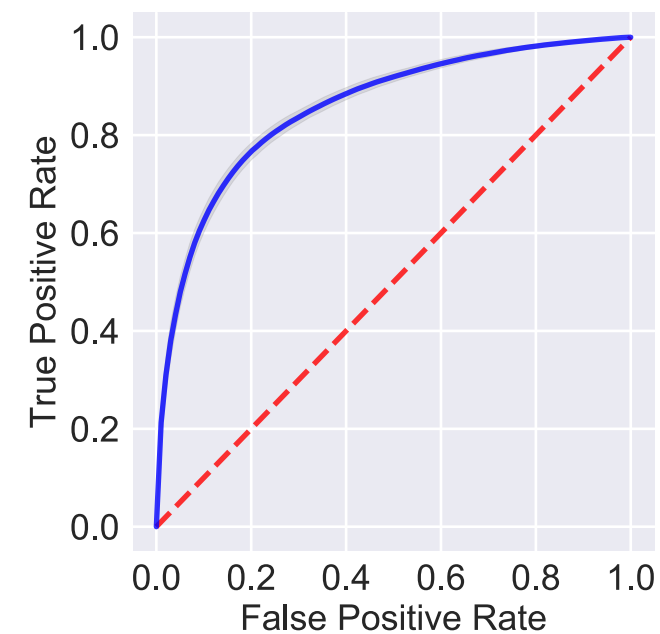

B

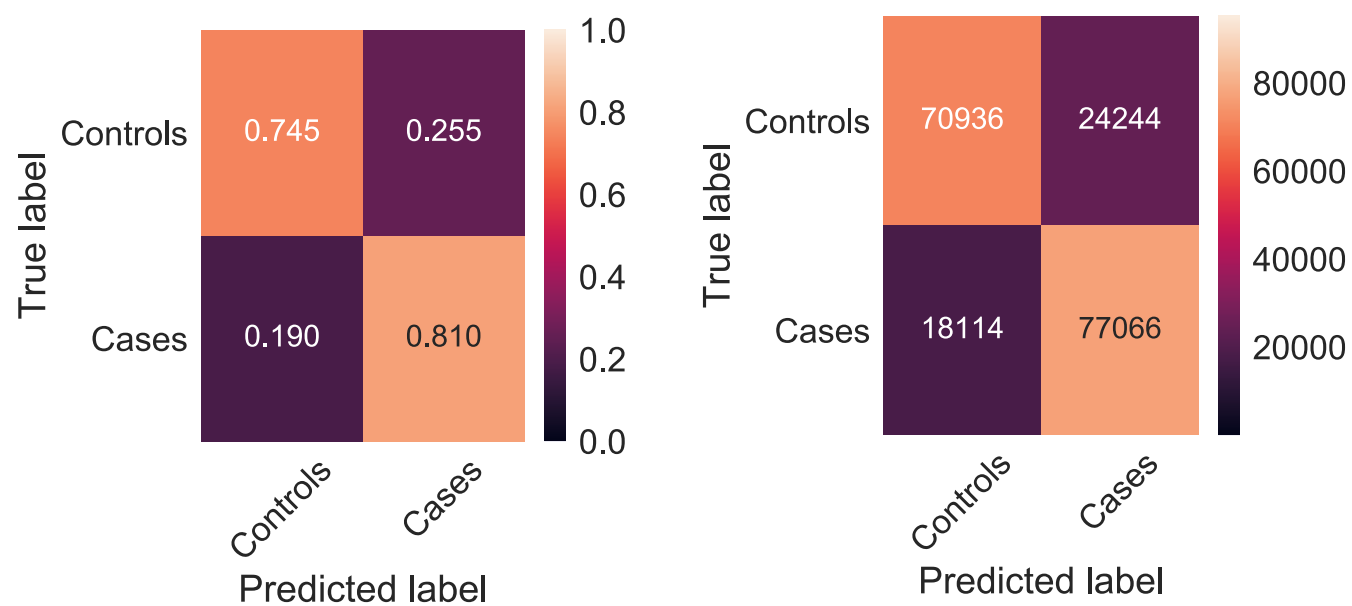

C

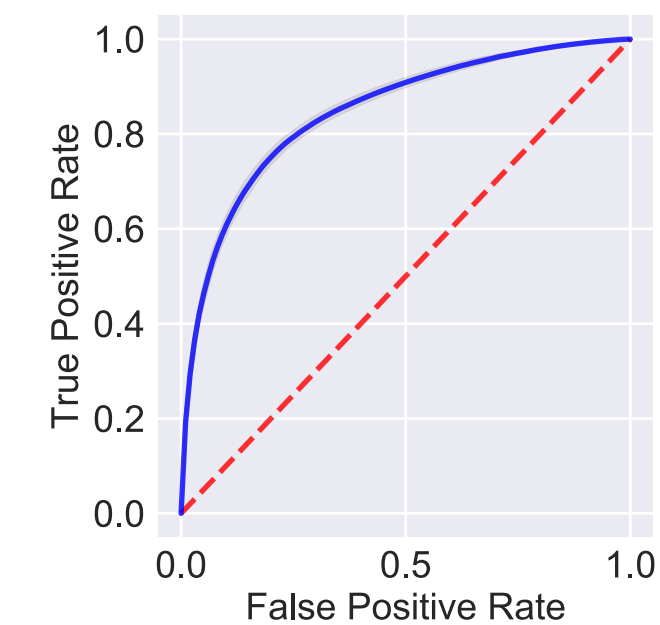

D

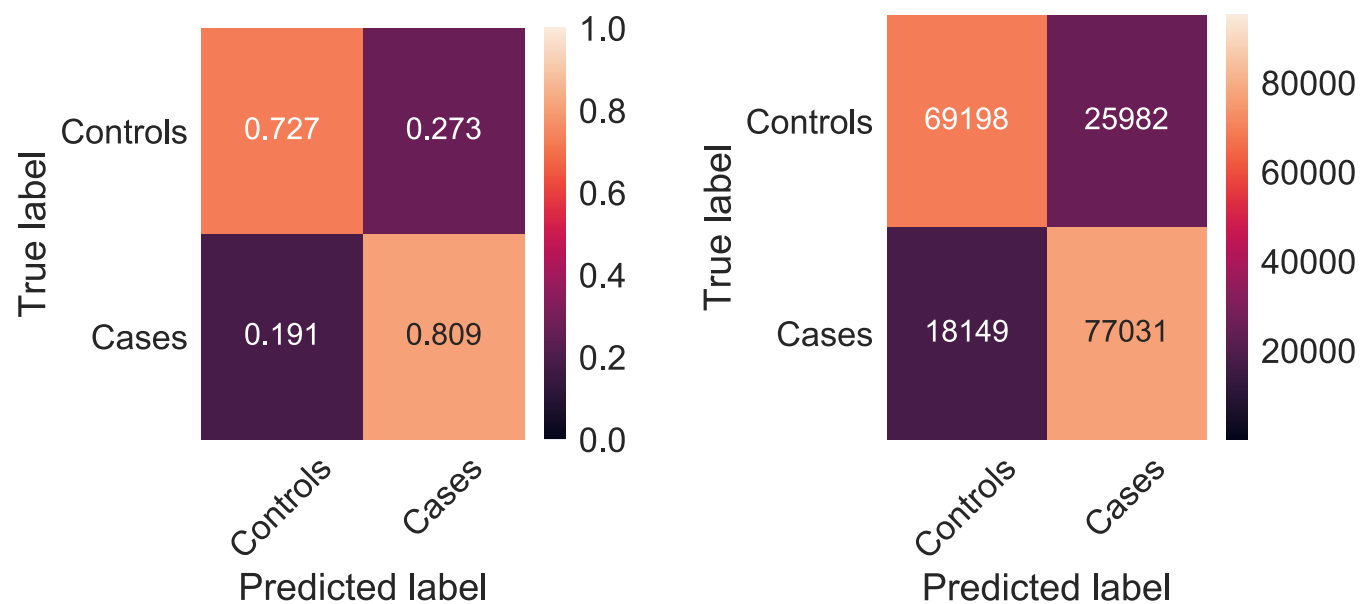

E

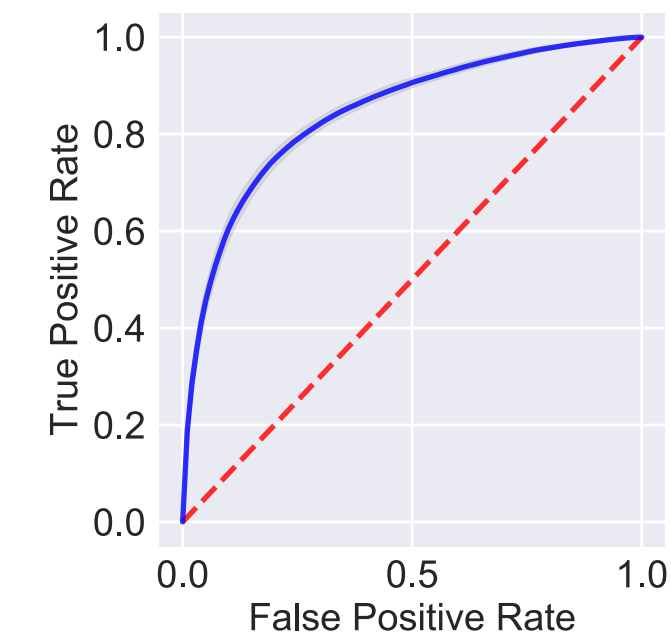

F

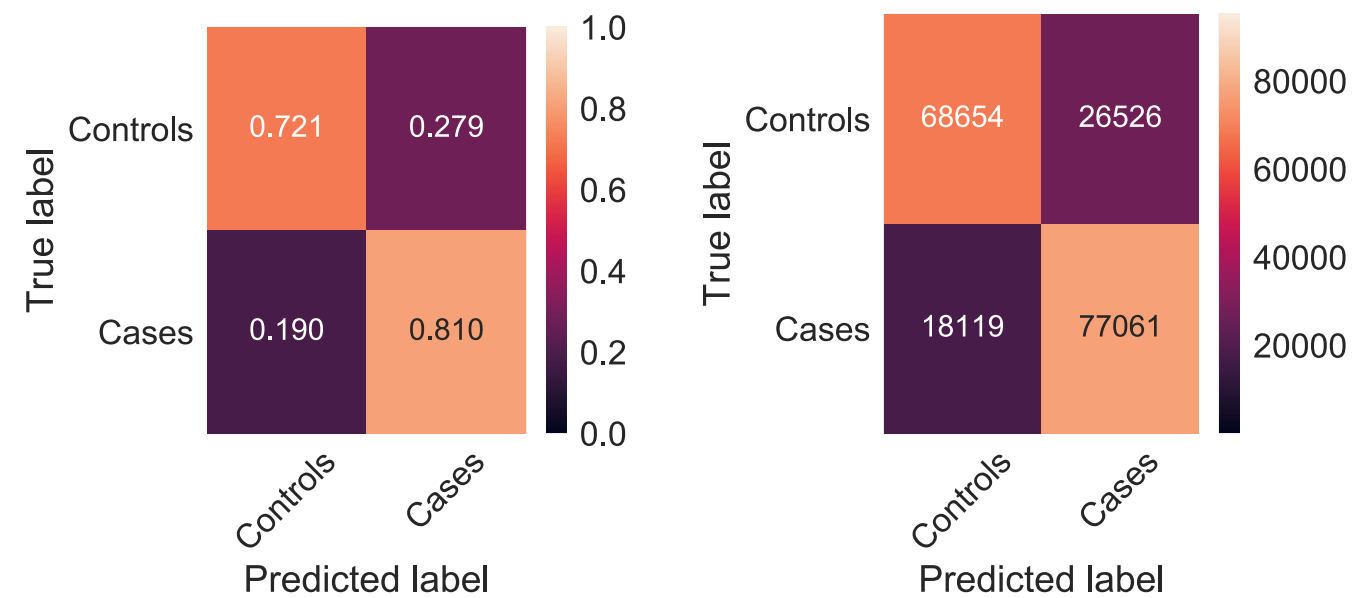

Supplement: Supplementary file 6 — Figure S6. Receiver operating characteristic curves, normalized, and non-normalized confusion matrices for classifiers using no imputation (A, B), imputation by the mean (C, D), and imputation by the median (E, F). (PDF 602 kb) [file 13040_2019_193_MOESM6_ESM.pdf]

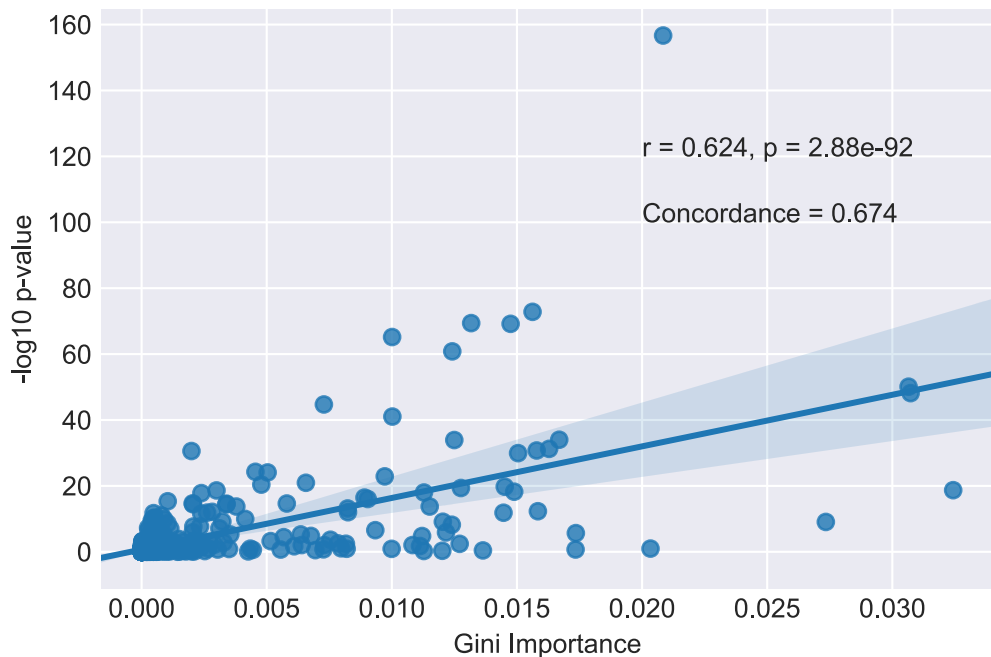

Supplement: Supplementary file 7 — Figure S7. Receiver operating characteristic curves, normalized, and non-normalized confusion matrices for labs and vitals from the 20 days prior to substance dependence diagnosis, classified using no imputation (A, B), imputation by the mean (C, D), and imputation by the median (E, F). (PDF 1335 kb) [file 13040_2019_193_MOESM7_ESM.pdf]

A

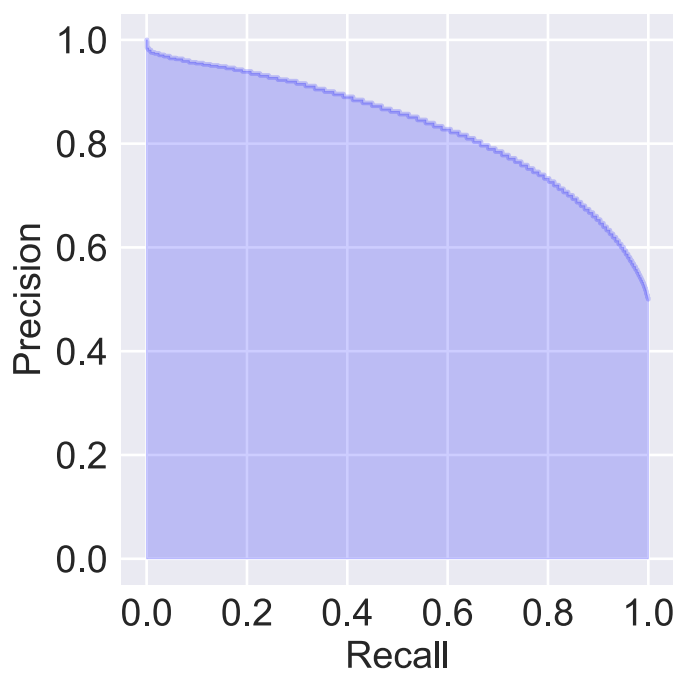

B

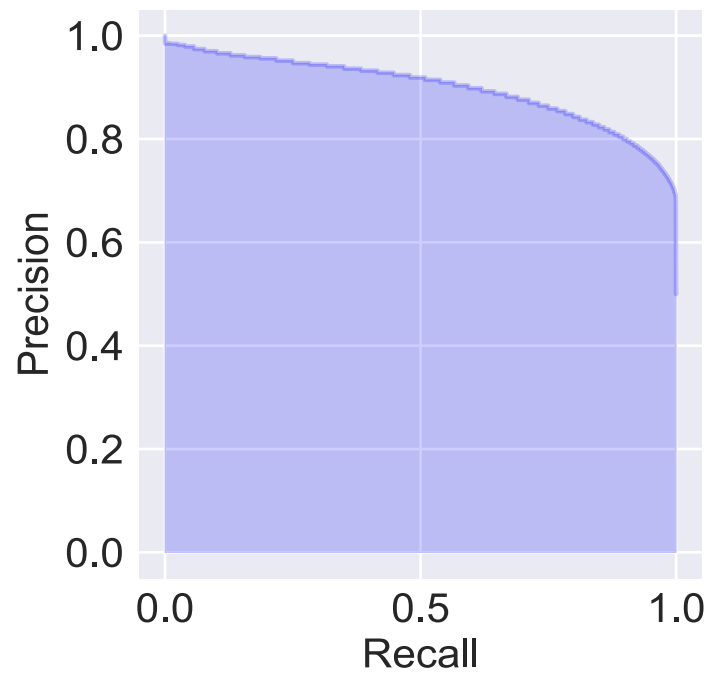

C

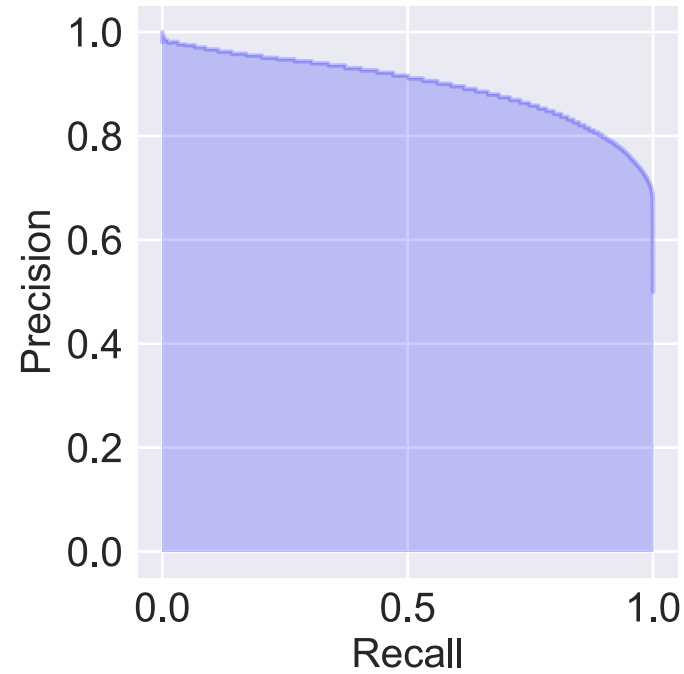

Supplement: Supplementary file 8 — Figure S8. Precision-recall curves for labs and vitals from the 20 days prior to substance dependence diagnosis, using classifiers with no imputation (A), imputation by the mean (B), imputation by the median (C). (PDF 199 kb) [file 13040_2019_193_MOESM8_ESM.pdf]

A

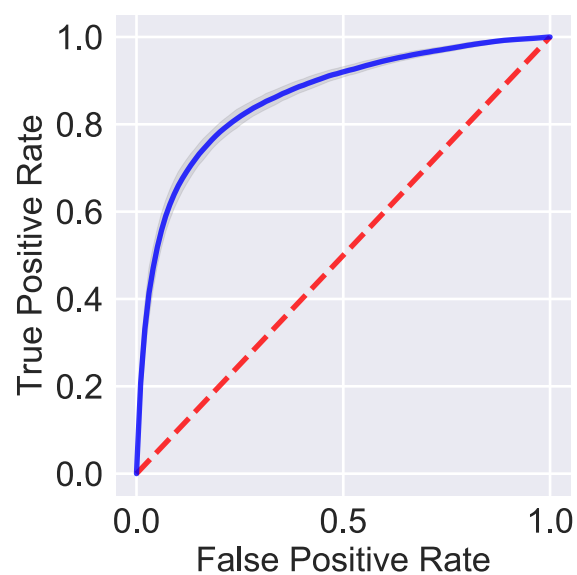

B

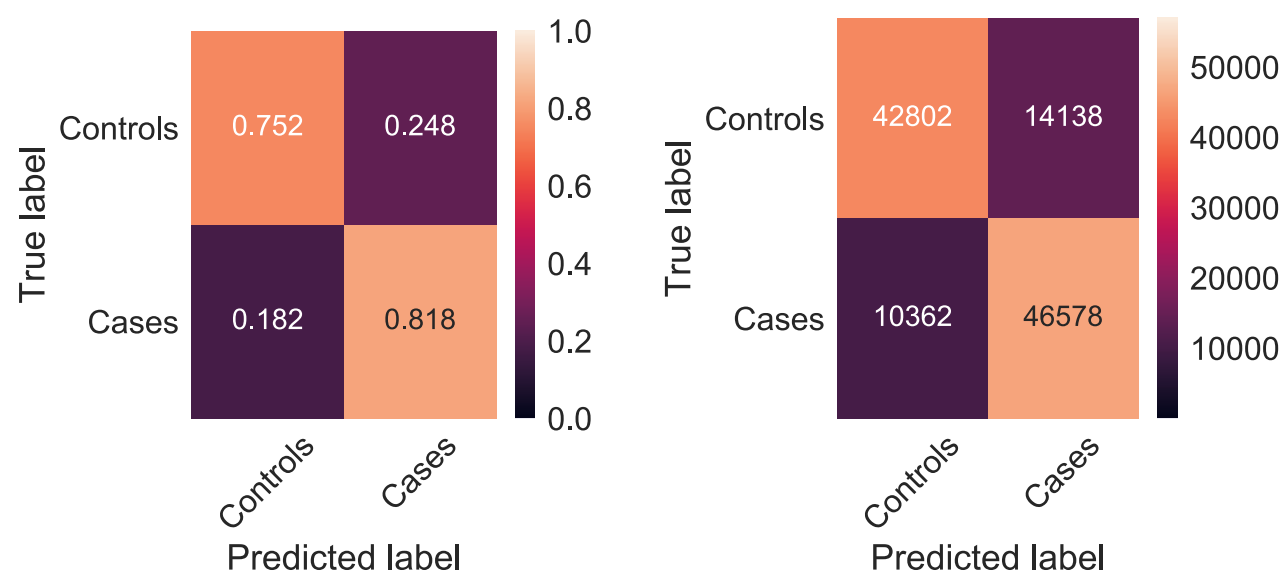

C

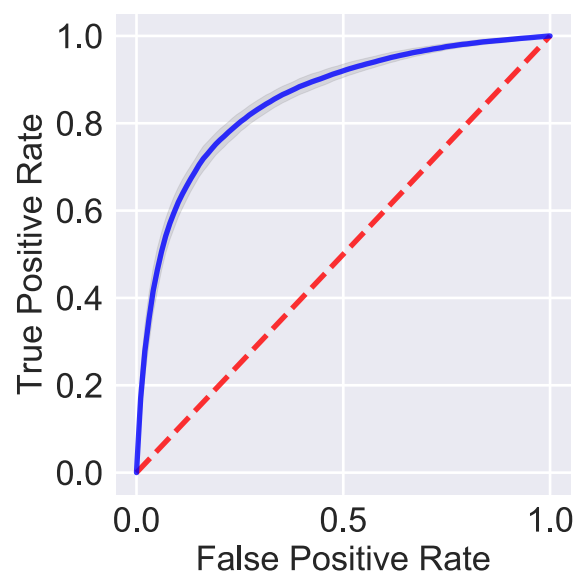

D

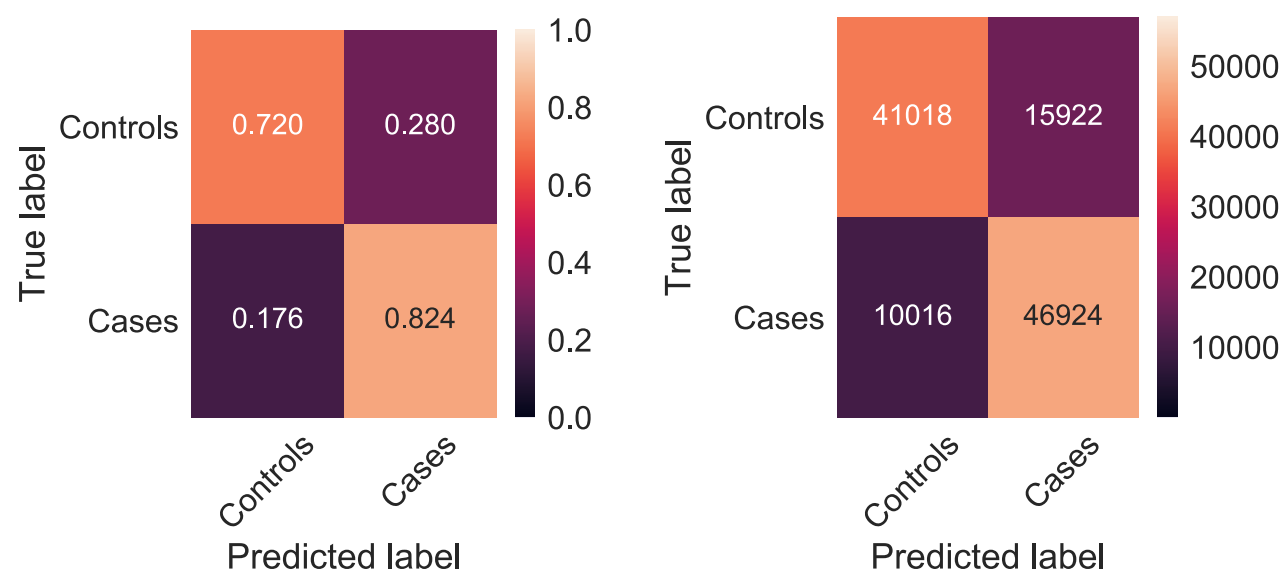

E

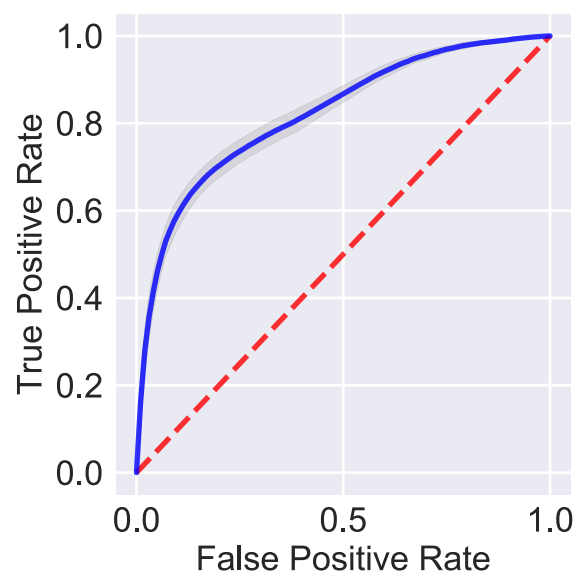

F

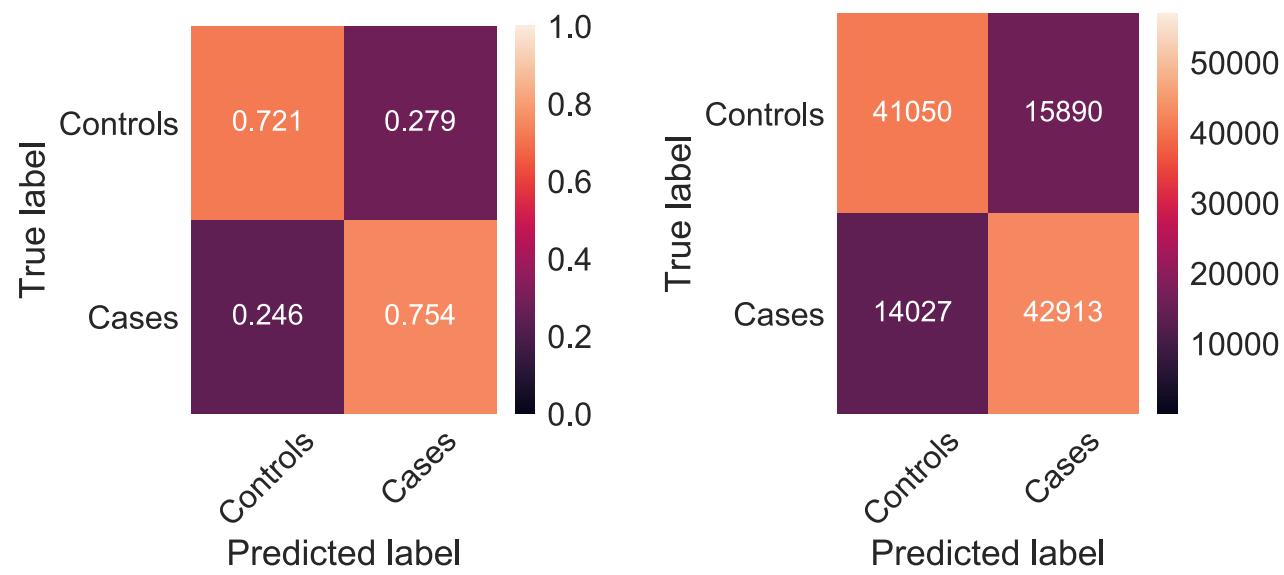

Supplement: Supplementary file 9 — Figure S9. Receiver operating characteristic curves, normalized, and non-normalized confusion matrices for diagnoses, prescriptions, and procedures from the 5 years prior to substance dependence diagnosis, classified using no imputation (A, B), imputation by the mean (C, D), and imputation by the median (E, F). (PDF 596 kb) [file 13040_2019_193_MOESM9_ESM.pdf]

A

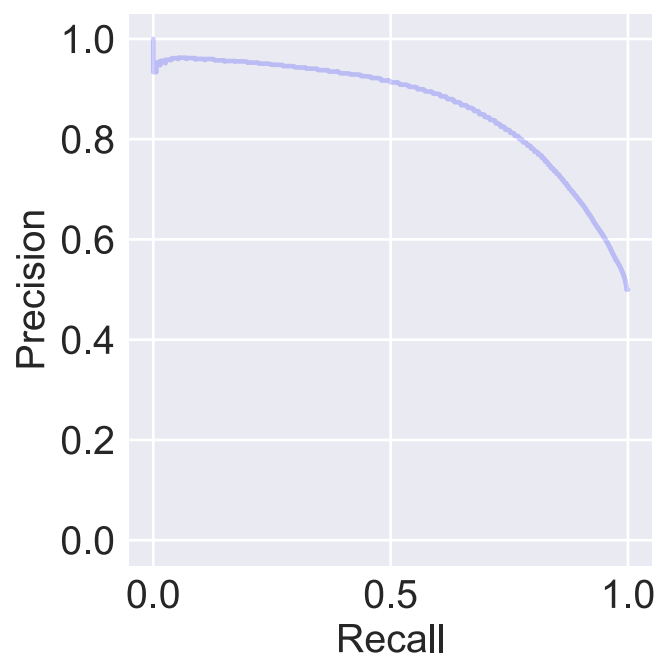

B

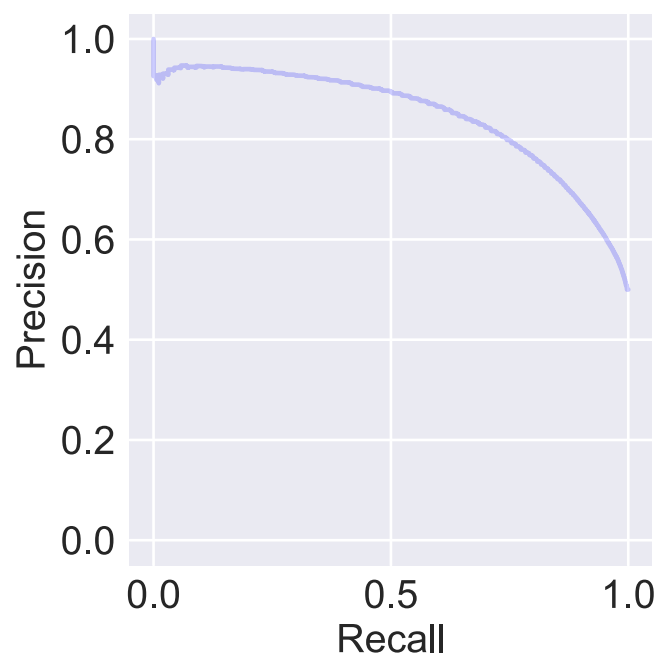

C

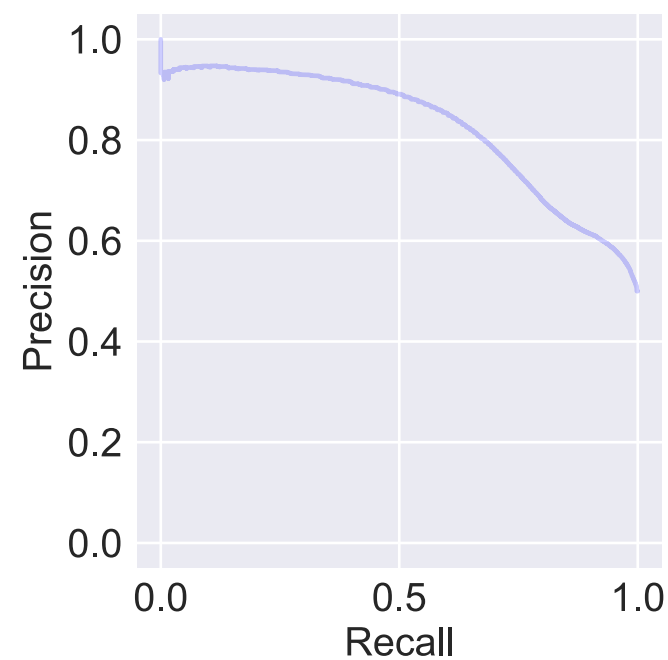

Supplement: Supplementary file 10 — Figure S10. Precision-recall curves for classifiers using diagnoses, prescriptions, and procedures from the 5 years to substance dependence diagnosis, with no imputation (A), imputation by the mean (B), imputation by the median (C). (PDF 212 kb) [file 13040_2019_193_MOESM10_ESM.pdf]

A

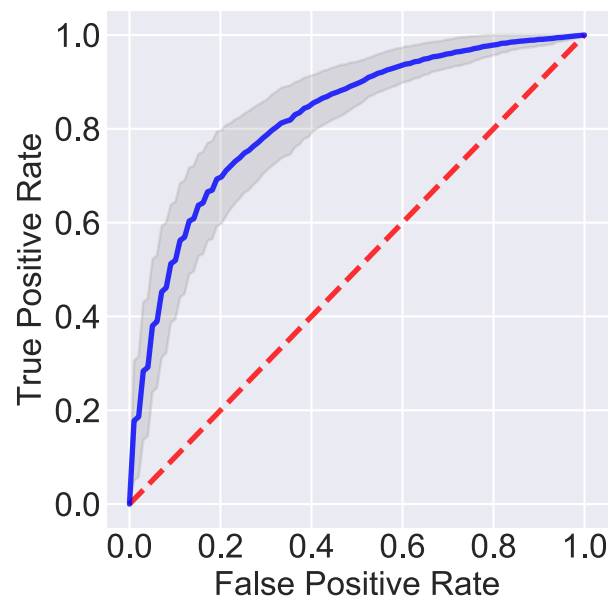

B

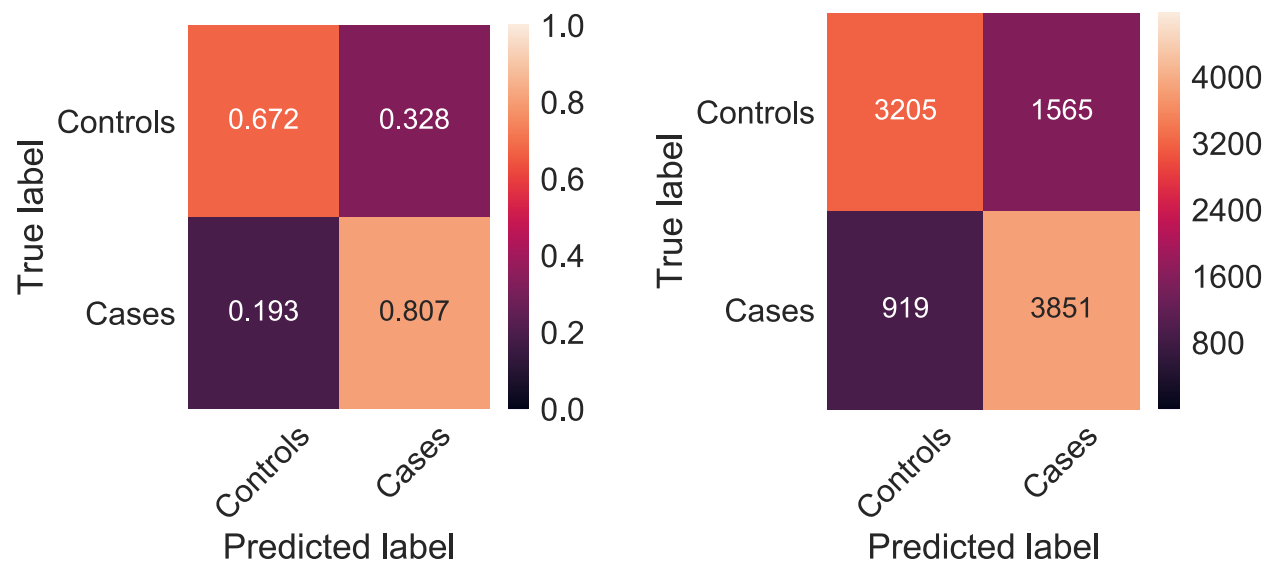

C

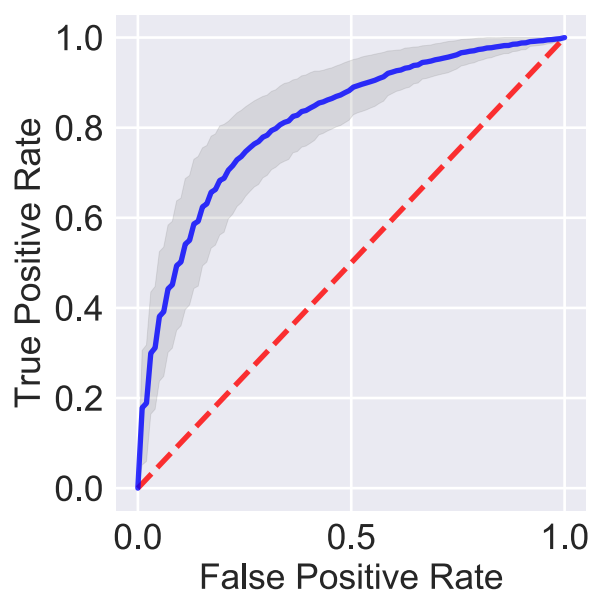

D

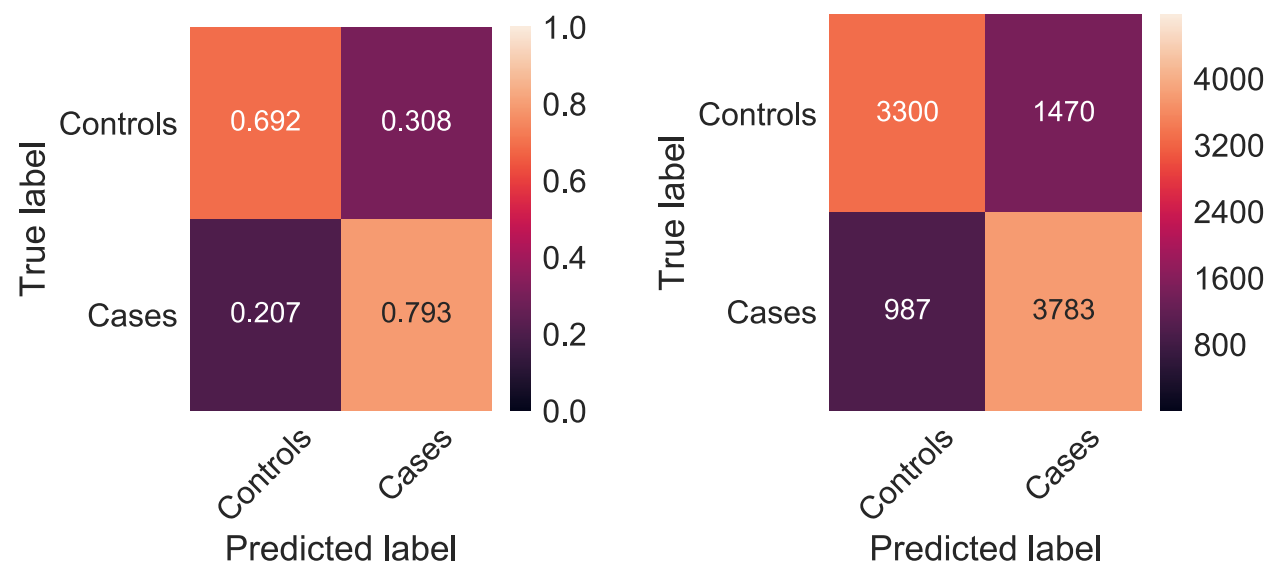

E

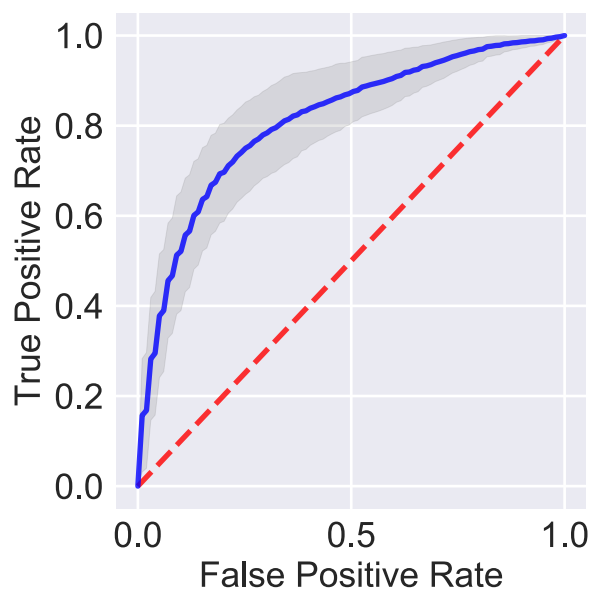

F

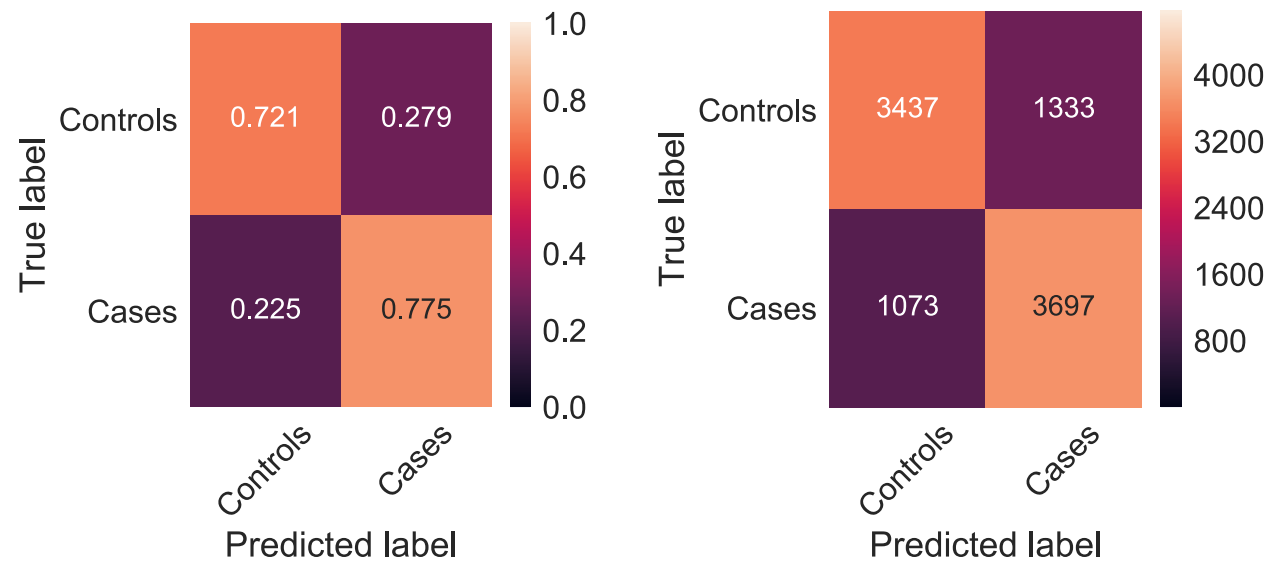

Supplement: Supplementary file 11 — Figure S11. Receiver operating characteristic curves, normalized, and non-normalized confusion matrices for lab tests and vital signs from the 6 months prior to opioid poisoning diagnosis, classified using no imputation (A, B), imputation by the mean (C, D), and imputation by the median (E, F). (PDF 597 kb) [file 13040_2019_193_MOESM11_ESM.pdf]
