# Supplementary material for: Predicting opioid dependence from electronic health records with machine learning
Source: BioData Min. 2019 Jan 29;12:3. doi: 10.1186/s13040-019-0193-0 (PMC6352440; doi:10.1186/s13040-019-0193-0)
Supplement: Supplementary file 5 — Figure S5. Raw values for cases and matched controls of the top 9 lab tests and vital signs by mean Gini importance. (PDF 174 kb) [file 13040_2019_193_MOESM5_ESM.pdf]

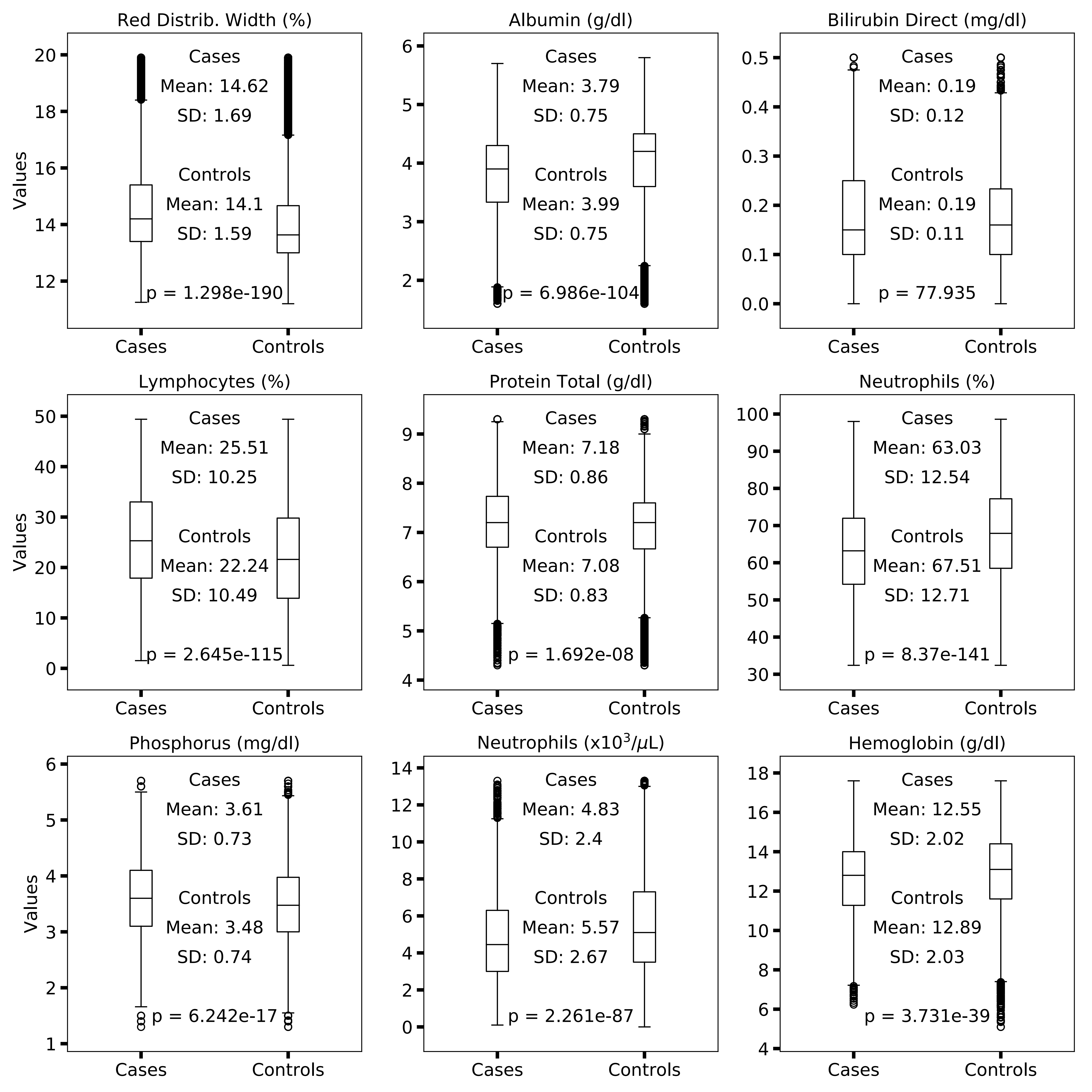
Red Distrib. Width (%)
Cases
Mean: 14.62
SD: 1.69
Controls
Mean: 14.1
SD: 1.59
p = 1.298e-190
Albumin (g/dl)
Cases
Mean: 3.79
SD: 0.75
Controls
Mean: 3.99
SD: 0.75
p = 6.986e-104
Bilirubin Direct (mg/dl)
Cases
Mean: 0.19
SD: 0.12
Controls
Mean: 0.19
SD: 0.11
p = 77.935
Lymphocytes (%)
Cases
Mean: 25.51
SD: 10.25
Controls
Mean: 22.24
SD: 10.49
p = 2.645e-115
Protein Total (g/dl)
Cases
Mean: 7.18
SD: 0.86
Controls
Mean: 7.08
SD: 0.83
p = 1.692e-08
Neutrophils (%)
Cases
Mean: 63.03
SD: 12.54
Controls
Mean: 67.51
SD: 12.71
p = 8.37e-141
Phosphorus (mg/dl)
Cases
Mean: 3.61
SD: 0.73
Controls
Mean: 3.48
SD: 0.74
p = 6.242e-17
Neutrophils ($x10^3/\mu L$)
Cases
Mean: 4.83
SD: 2.4
Controls
Mean: 5.57
SD: 2.67
p = 2.261e-87
Hemoglobin (g/dl)
Cases
Mean: 12.55
SD: 2.02
Controls
Mean: 12.89
SD: 2.03
p = 3.731e-39
Values
Cases
Controls
